# Happiness predicts compliance with preventive health behaviours during Covid-19 lockdowns

**DOI:** 10.1038/s41598-023-33136-9

**Published:** 2023-05-17

**Authors:** Christian Krekel, Sarah Swanke, Jan-Emmanuel De Neve, Daisy Fancourt

**Affiliations:** 1 London School of Economics and Political Science, London, UK; 2 Max Planck Institute for Human Development, Berlin, Germany; 3 University of Oxford, Oxford, UK; 4 University College London, London, UK

**Keywords:** Disease prevention, Public health, Quality of life

## Abstract

To combat the public health crisis of Covid-19, governments and public health officials have been asking individuals to substantially change their behaviours for prolonged periods of time. Are happier people more willing to comply with such measures? Using independent, large-scale surveys covering about 79,000 adult respondents across 29 countries, including longitudinal data from the UK, we find that life satisfaction predicts compliance with preventive health behaviours during Covid-19 lockdowns, especially the number of weekdays stood at home (β = 0.02, *p* < 0.01). The association is stronger for higher levels of life satisfaction (e.g. β = 0.19, *p* < 0.01, 7 on a 0-to-10 scale). Lower life satisfaction, on the contrary, predicts lower compliance (e.g. β = 0.02, *p* > 0.10, 2 on a 0-to-10 scale). We explore risk-avoidance and pro-social motivations for this relationship, and find suggestive evidence that people who are older or have certain medical preconditions seem to be behave in line with risk-avoidance, whereas motivations of people who are less at risk of Covid-19 seem more mixed. While it is difficult to estimate the relationship between life satisfaction and compliance behaviour due to potential confounders and unobserved heterogeneity, our findings suggest that life satisfaction is important, both for complying with preventive health measures and as a policy end in itself.

## Introduction

There is growing, interdisciplinary evidence in the social science literature spanning economics, psychology, and epidemiology that supports the predictive power of subjective wellbeing for subsequent behaviour. For example, people who are happier or have higher life satisfaction have been shown to be more productive^1,2^, to live longer^3–7^, and to be more likely to vote for an incumbent government, an association that holds even after controlling for socio-economic factors^8–10^. Most recently, it has been shown that feelings have greater predictive power for subsequent actions than a combined set of economic and social variables, and that there is an inverse relationship between positive feelings and subsequent “get-me-out-of-here” actions in various life domains (e.g. moving house, partner separation, changing job, or moving to hospital)^11^.

We contribute to this growing body of evidence by studying whether life satisfaction predicts compliance with preventive health behaviours, complementing existing evidence on compliance that looks primarily at socio-demographic, economic, or attitudinal factors^12–14^, incidental factors like meteorological conditions^15^, or the way information on health risks and others’ behaviours is provided^16^. In particular, we look at the unique setting of Covid-19 lockdowns, in which governments and health officials around the world have been asking their citizens to substantially change their behaviours for prolonged periods of time, by practising physical distancing and staying at home, amongst others. Are people who are more satisfied with their lives more willing to comply with such measures?

The answer to this question is not *ex-ante* clear. The psychological literature focuses mostly on changes in mood and affect, which are considered to be more temporary states, whereas overall life satisfaction—as a global, cognitive measure of subjective wellbeing—is defined as evaluations of one’s life on a longer time scale^17^. We are the first to study the association of this longer-term measure with preventive health behaviours.

When considering mood and affect, the two dominant psychological theories make contradictory predictions about compliance, regardless of whether the underlying motivation is risk-avoidance or pro-social behaviour. The *affect infusion model*^18^ predicts that people in positive affect show less compliance, because positive affect makes people perceive risky prospects more favourably (in the case of risk-avoidance) or because it creates more internally oriented mental processing that promotes selfishness (in the case of pro-social behaviour)^19,20^. On the contrary, the *mood maintenance model*^21^ predicts that people in positive affect show more compliance in the case of risk avoidance, as positive affect makes people more loss averse^22^. In the case of pro-sociality, predictions can even go either way: people in positive affect may show more compliance if compliance promotes their self-image, makes them appear more favourable in social comparisons, or promotes good feelings more generally. However, people may show less compliance if complying becomes too unpleasant^23^. Table 1 summarises these different theoretical predictions. Importantly, these predictions are mostly based on lab experiments which experimentally manipulate incidental mood, rather than observing a person’s overall life satisfaction in a naturalistic setting.

**Table 1:** Theoretical predictions about effect of mood on compliance.

Compliance behaviour	Affect infusion model	Mood maintenance model
Risk-avoidance motivation	Positive mood and affect decrease compliance	Positive mood and affect increase compliance
	Mechanism: change in shape of probability-weighting function (less weight attached to risky prospects)^18,19^	Mechanism: loss aversion (people want to retain positive mood and affect)^21,22^
Pro-social motivation	Positive mood and affect decrease compliance	Positive mood and affect increase/decrease compliance
	Mechanism: change in internally versus externally oriented mental processing (more internally oriented mental processing promotes selfishness)^20^	Mechanism: increase in compliance if promotion of self-image, favourable appearance in social comparisons, good feelings more generally; decrease in compliance if compliance becomes too unpleasant^23^

To explore the association between overall life satisfaction and preventive health behaviours in a naturalistic setting, and to test whether this association aligns with either one of these psychological theories, we exploit Covid-19 lockdowns as a large-scale, quasi-natural experiment. We primarily look at self-reported health behaviours, both between and within countries. Covid-19 lockdowns yield ideal quasi-experimental conditions: they require a homogeneous set of health behaviours for a limited amount of time from almost everybody in the population, with few exceptions. However, people differ substantially in terms of health consequences when catching the virus: the case-fatality ratio increases exponentially from age 60 onwards^24,25^, and people with certain medical preconditions—high blood pressure, diabetes, and heart and lung disease—are at particular risk^26,27^. We further exploit these age and health gradients to shed light on underlying motivations for their health behaviours. The focus of our investigation is the first set of “hard” lockdowns worldwide: in early 2020, when the virus was still novel, there had been little learning in terms of health behaviours, and vaccinations had not yet been developed. The quasi-experimental conditions we seek to exploit for our investigation are the cleanest during this period.

In Study 1, we look at whether present individual life satisfaction predicts self-reported individual compliance with preventive health behaviours in various domains. Here, we use cross-sectional data from the Imperial College London-YouGov Covid-19 Behavioural Tracker, on around 39,000 respondents from 330 regions in 29 countries. In our main Study 2, we focus on the Covid-19 lockdown in a single country, namely the UK. We use the University College London Covid-19 Social Survey, a panel of almost than 40,000 individuals, which starts just before the first “hard” lockdown (March 23, 2020) and includes weekly data on participants across the entire lockdown period (up until May 10, 2020). We apply individual fixed effects and dynamic panel data estimators to look at how present individual life satisfaction predicts self-reported individual compliance longitudinally, within individuals. We then exploit the age and health gradients of respondents to explore potential motivations underlying compliance behaviour.

The Supplementary Materials include yet another study that looks at whether regional life satisfaction in 2019 predicts regional compliance with Covid-19 measures, using nationally representative cross-sectional data on about 50,000 respondents from the Gallup World Poll (892 regions in 49 countries), and measuring compliance objectively using changes in geographical mobility, obtained from Google Maps-recorded visits to, or time spent in, various locations. The results of this additional study, which are less robust than the studies presented in the main text, corroborate our finding that life satisfaction is positively associated with compliance with Covid-19 measures.

To ensure clean quasi-experimental conditions, we restrict our sample to observations where the “stringency” of lockdown measures is equal to or greater than 72.48 (obtained from the Oxford COVID-19 Government Response Tracker), corresponding to the stringency observed during the first “hard” UK lockdown, which is the focus of our main Study 2. We omit individuals who have not had Covid-19 (to their knowledge) and who are non-key workers, since these individuals have lower risk profiles when (re-)contracting Covid-19 (in case of having contracted it before) and were subject to different regulations (in case of key workers). Our results are robust to lifting these restrictions.

Our paper contributes to the literature on the determinants of compliance with preventive health behaviours during lockdowns, which had been a completely new experience in the lives of most people, including, amongst others, socio-demographic factors^12,28–30^, beliefs and expectations^31–33^, trust and social capital^13,34–36^, political partisanship^37–39^, and the type of state and local policies, including state-of-emergency declarations, stay-at-home restrictions, or industry-specific restrictions^40^. Interestingly,^40^ find that private, self-regulating behaviour explains more than three-quarters of the decline in foot traffic in most industries. By pointing towards the potentially mediating role of people’s overall life satisfaction for risk-taking and compliance, our findings may help reconcile contradictory evidence on risk-avoidance and compliance, in particular that perceived personal health risks associated with Covid-19 are found to *decrease* with age^41^ yet that regions with less risk-takers and larger older population are found to *increase* their time staying home during strict lockdowns^42^. Finally, our paper contributes to the literature on the relationship between wellbeing, affect, and behaviour more generally, adding to evidence on how wellbeing and affect are associated with risk-taking (see^43,44^ or^45^, for example) and pro-social behaviour (see^46–48^ or^49^, for example).

### Ethical approval

This research uses secondary data only. We confirm that all methods were carried out in accordance with relevant guidelines and regulations. Moreover, all experimental protocols were approved by the data custodians and informed consent was obtained from all subjects prior to secondary data collection.

## Study 1: cross-sectional evidence on individual life satisfaction and self-reported individual compliance

Throughout the world, in the early months of 2020, different countries introduced lockdowns to contain the spread of Covid-19. They were introduced in different places at different points in time, with variation in terms of severity, depending on local characteristics, as well as number of confirmed cases and deaths. It is estimated that, by late March 2020, more than 100 countries had introduced either full or partial lockdowns^50^. We exploit this variation between countries across the world in our first empirical investigation. In particular, we study whether present individual life satisfaction predicts individual compliance with preventive health behaviours during Covid-19 lockdowns, contemporaneously.

### Data and methods

We use cross-sectional data from the Imperial College London-YouGov Covid-19 Behavioural Tracker, an international survey which has been running in 29 countries since April 2020 and includes data on about 39,000 respondents’ life satisfaction alongside individual and household characteristics, with a particular focus on behaviour in response to Covid-19 and lockdown measures^51^. The Supplementary Materials on Study 1 include a list of countries with the country-specific observation periods. We merge these data with the Oxford COVID-19 Government Response Tracker to include the stringency of lockdown measures and confirmed cases and deaths at the country level^52^.

Our main outcome is the extent to which a respondent reports to be willing to self-isolate for seven days if asked by a healthcare professional or public health authority. It is obtained from a single-item five-point Likert scale ranging from one (“very willing”) to five (“very unwilling”). We dichotomise this item to equal one if a respondent is “very willing” or “somewhat willing” to comply, zero otherwise. Other outcomes include the frequency of complying with 20 common preventive health behaviours, such as wearing a face mask, washing hands, or using sanitiser. These are obtained from single-item five-point Likert scales ranging from one (“always”) to five (“not at all”). We again dichotomise these items to equal one if a respondent is “always” or “frequently” complying, zero otherwise. We then create an overall index of compliance with preventive health behaviours, by standardising each item and calculating a weighted sum, where we use the inverse of the variance–covariance matrix of the standardised items as weights. This ensures that highly correlated items, which contain little new information, receive less weight.

Our variable of interest is life evaluation (also known as the *Cantril ladder*; see^53^). It is obtained from a single-item eleven-point Likert scale which asks respondents to imagine themselves on a ladder with steps numbered from zero at the bottom to ten at the top, where zero represents the worst possible and ten the best possible life. In practice, life evaluation and life satisfaction are often seen as equivalent, so in what follows we refer to it as *life satisfaction* for simplicity.

We regress self-reported compliance behaviour on respondents’ present life satisfaction during Covid-19 lockdowns. Our analysis is restricted to periods where the stringency of lockdown measures is (at least) as high as that observed during the first “hard” lockdown in the UK, to ensure comparability between Studies 1 and 2. Moreover, we restrict our sample to individuals who (and whose household cohabitants) report to have never been tested for and to have never had any symptoms of Covid-19, to ensure that individuals in our sample have similar risk profiles in case of contracting Covid-19. Our results continue to hold when lifting these restrictions (see Supplementary Materials Tables S.1.3 and S.1.4). Our sample includes data on between 12,520 and 38,910 adult respondents (depending on outcome) in 29 countries. The Supplementary Materials on Study 1 include more details on methods, with Table S.1.5 showing summary statistics.

Note that, although we are controlling for observable characteristics at the individual level (i.e. age, gender, employment status, and the number of adults and children in the household), the log daily numbers of confirmed Covid-19 cases and deaths at the country level, and fixed effects (i.e. weekday, calendar week, and country fixed effects), our estimation relies on between-individual variation in life satisfaction and compliance behaviour, due to the cross-sectional nature of our data. That said, we are unable to control for time-invariant unobservable heterogeneity (e.g. personality traits possibly related to compliance such as agreeableness in the Big-5 personality inventory) or time-invariant, potentially omitted observables at the individual level. Our obtained coefficients should, therefore, be interpreted as associations and potentially upper-bound estimates of the true association between compliance behaviour and life satisfaction. A potentially important omitted observable is income, which is not asked about in the Imperial College London-YouGov Covid-19 Behavioural Tracker and which may contribute to an overestimate of the association. We are trying to mitigate this issue by controlling for country fixed effects (i.e. differences in income levels at the country level), by using a short observation period (i.e. about two months in each country, over which income levels should be relatively stable), and by restricting our final sample to individuals who are subject to a homogeneous policy response to Covid-19 (which may limit the choices people have as a function of their incomes).

### Findings

We find that higher levels of life satisfaction of respondents are associated with greater contemporaneous self-reported compliance behaviour during Covid-19 lockdowns (Supplementary Materials Table S.1.1). Associations are highly significant though rather small in size: a one-point increase in life satisfaction is associated with an increase in respondents’ self-reported willingness to comply with recommendations of about one percentage point (β = 0.008, *p* < 0.01). Similarly, a one-point increase in life satisfaction is associated with a 1.3% standard-deviation increase in our overall index of compliance with preventive health behaviours (β = 0.013, *p* < 0.01). Looking at the different health behaviours in more detail, Supplementary Materials Table S.1.2 shows that life satisfaction has consistent, significant positive associations with self-reported health behaviours across various domains, including washing hands (β = 0.004, *p* < 0.01) or using sanitisers (β = 0.01, *p* < 0.01), avoiding shopping (β = 0.004, *p* < 0.05), or avoiding transit (β = 0.004, *p* < 0.01) and crowded areas (β = 0.003, *p* < 0.05).

When including a (country-specific) dummy that is one if a respondent has already been subjected to lockdown (with a stringency equal to or greater than 72.48, corresponding to the stringency during the strict lockdown in the UK) for more than one week at the time of the interview, and zero else, we find that our results remain qualitatively the same as before (upon request).

## Study 2: longitudinal evidence on individual happiness and self-reported individual compliance

We now focus on how present individual life satisfaction predicts self-reported individual compliance with preventive health behaviours longitudinally, within individuals, using the example of a single country: the UK. The first “hard” UK lockdown officially started on Monday, March 23, 2020, after a prime-time television broadcast of then Prime Minister Boris Johnson appealing to the general public to stay at home. This came after the Prime Minister had already ordered all pubs, cafés, restaurants, and gyms to close, and after the Chancellor of the Exchequer had announced that the public purse would meet 80% of the wages of employees who could not do their work under lockdown. At this point, many people were already voluntarily staying at home. As was the case elsewhere, the nation-wide lockdown was a curfew under which people should leave their homes only for several reasons, including essential shopping or one outdoor exercise per day. It effectively ended on Sunday, May 10, 2020, when the Prime Minister announced a three-step roadmap out of lockdown. As of the following day, certain non-essential workers in England were encouraged to go back to work and unlimited outdoor exercise was allowed (still subject to physical distancing). This lockdown was the first out of several (including two subsequent “hard” ones and periods with fluctuating intensities of restrictions, partly with regional variation), but it was at a time when the virus was still novel, there had been little learning in terms of health behaviours, and vaccinations had not yet been developed or on the roll-out. The quasi-experimental conditions for our analysis are, therefore, cleanest during this first nation-wide lockdown period.

### Data and methods

We use longitudinal data from the University College London Covid-19 Social Survey, a weekly online panel of more than 70,000 individuals in England, Wales, Scotland, and Northern Ireland, which starts on March 21, 2020. We limit our analyses to the period between March 20 and May 10, 2020. We further restrict it to respondents who identify as non-key workers (thereby excluding essential workers, including those in health or social care, who were not under lockdown); respondents who report to have never had Covid-19 themselves; and respondents who reported to have never had contact with people who had contracted the virus (as these have different risk profiles when contracting Covid-19). Again, our results continue to hold when lifting these restrictions (see Supplementary Materials Table S.2.6). The stringency of lockdown measures, and log daily numbers of confirmed Covid-19 cases and deaths are again obtained from the Oxford COVID-19 Government Response Tracker.

We look at three outcomes of self-reported compliance with preventive health behaviours. These are (i) the number of days during the past seven days the respondent reports to have stayed at home and not left the house (zero-to-seven Likert scale); and (ii) whether the respondent reports to be either fully, partially, or not at all self-isolating at present (binary indicators). Fully self-isolating means not leaving home at all, partially self-isolating means complying with stay-at-home recommendations and leaving home only for necessary activities, and not at all self-isolating means not complying with stay-at-home recommendations and living life as normally as possible. Finally, we look at (iii) the degree to which the respondent reports to comply with government recommendations (one-to-seven Likert scale). Our preferred outcome is the number of days during the past seven days the respondent reports to have stayed at home, which may be least susceptible to social desirability tendencies: it is an unambiguous, past-oriented behavioural measure that does not commingle behaviours with attitudes, neither implicitly nor explicitly. Importantly, it was never forbidden to leave the house at any point in the UK. In fact, respondents could always leave for essential shopping or physical exercise, allowing them to report their true behaviour without feeling sanctioned.

Our variable of interest is life satisfaction, which is obtained from a single-item eleven-point Likert scale asking respondents: “Overall, in the past week, how satisfied have you been with your life?” Answers ranged from zero (“not at all”) to ten (“completely”). We adjusted this variable to account for its retrospective nature, by making life satisfaction concurrent to the outcomes in the past week.

We start with pooled OLS regressions which do not account for the panel dimension of our data (see Supplementary Materials Study 2 Eq. 2.1), and then make our models successively more restrictive: first, we account for individual fixed effects (time-invariant unobservables and observables at the individual level) using both within-transformation and first-differences (Eq. 2.2). Then, we allow for *lagged* compliance in the past (up to a second lag as we find compliance behaviour to follow an autoregressive process of order two, i.e. an AR(2) process) to influence *current* compliance behaviour, applying dynamic panel data models using Arellano-Bond estimators with generalised method of moments (GMM) estimation (Eq. 2.3). In these latter models, past compliance behaviour is held constant to explain current compliance behaviour jointly with current levels of life satisfaction, thereby exploiting the dynamic structure of the data to reduce concerns about reverse causality. Our sample includes data on between 28,897 and 34,378 adult respondents (depending on outcome and model). The Supplementary Materials on Study 2 include more details on methods, with Table S.2.7 showing summary statistics.

Unlike Study 1, we are now controlling not only for (a much wider set of) observable characteristics at the individual level, the log daily numbers of confirmed Covid-19 cases and deaths at the country level (i.e. England, Wales, Scotland, and Northern Ireland), and weekday and country fixed effects, but also for individual fixed effects and (in our most restrictive model) lagged compliance behaviour. Our estimation, therefore, relies on within-individual variation in life satisfaction and compliance behaviour. That said, we are controlling for time-invariant unobservable heterogeneity as well as time-invariant, potentially omitted observables at the individual level. While our coefficients should still be interpreted as associations, they are residuals of what is left over when everything else (both observable and unobservable) is accounted for, including confidence in government and the public health system, measures of current depression and anxiety, and lagged compliance in the previous week. They can thus be interpreted as close to the direct, net association of life satisfaction with compliance behaviour. Note that we routinely control for household income (measured in income brackets) throughout our regressions.

### Findings

Table 2 shows our main findings. We find that individuals who report higher levels of life satisfaction are significantly more likely to report higher levels of compliance with preventive health behaviours.

**Table 2:** Life satisfaction and self-reported compliance (University College London Covid-19 Social Study, UK, Year 2020).

	Static panel data estimation			Dynamic panel data estimation
	Pooled OLS	FE	FD	Arellano-Bond
	(1)	(2)	(3)	(4)
Panel A: number of weekdays staying home				
Life satisfaction	0.0623***	0.0184***	0.0211***	0.0202***
	(0.0064)	(0.0056)	(0.0059)	(0.0051)
Hansen test *P* value	–	–	–	0.744
AR(1), AR(2), AR(3) *P* values	–	–	–	0.000, 0.000, 0.110
Observations	131,088	131,088	99,403	100,850
Individuals	34,136	34,136	28,897	29,753
R^2^ (GMM: F)	0.086	0.006	0.005	F(77, 29,752) = 256.99
Panel B: fully isolating				
Life satisfaction	− 0.0001	− 0.0045***	-0.0009	0.0009
	(0.0007)	(0.0007)	(0.0006)	(0.0007)
Hansen test *P* value	–	–	–	0.540
AR(1), AR(2), AR(3) P-Values	–	–	–	0.000, 0.004, 0.987
Observations	132,703	132,703	102,327	104,020
Individuals	34,046	34,046	29,040	29,967
R^2^ (GMM: F)	0.078	0.036	0.001	F(77, 29,966) = 105.55
Panel C: partially isolating				
Life Satisfaction	0.0012	0.0039***	0.0017**	0.0003
	(0.0007)	(0.0010)	(0.0008)	(0.0008)
Hansen test *P* value	–	–	–	0.793
AR(1), AR(2), AR(3) *P* values	–	–	–	0.000, 0.000, 0.618
Observations	132,703	132,703	102,327	104,020
Individuals	34,046	34,046	29,040	29,967
R^2^ (GMM: F)	0.057	0.026	0.016	F(77, 29,966) = 113.72
Panel D: not isolating				
Life satisfaction	− 0.0011**	0.0006	− 0.0008	− 0.0013***
	(0.0004)	(0.0007)	(0.0006)	(0.0004)
Hansen test *P* value	–	–	–	0.950
AR(1), AR(2), AR(3) *P* values	–	–	–	0.000, 0.000, 0.929
Observations	132,703	132,703	102,327	104,020
Individuals	34,046	34,046	29,040	29,967
R^2^ (GMM: F)	0.121	0.132	0.032	F(77, 29,966) = 37.19
Panel E: complying with recommendations				
Life satisfaction	0.0154***	0.0036***	0.0018	0.0160
	(0.0015)	(0.0014)	(0.0015)	(0.0124)
Hansen test *P* value	–	–	–	0.677
AR(1), AR(2), AR(3) P values	–	–	–	0.823, 0.791, 0.601
Observations	136,385	136,385	107,278	108,298
Individuals	34,378	34,378	29,548	29,713
R^2^ (GMM: F)	0.105	0.013	0.013	F(77, 29,712) = 32.26
Controls	Yes	Yes	Yes	Yes
Area fixed effects	Yes	Yes	Yes	Yes
Country fixed effects	Yes	Yes	Yes	Yes
Week-day fixed effects	Yes	Yes	Yes	Yes
Week fixed effects	No	No	No	No
Individual fixed effects	No	Yes	Yes	Yes

See Study 2 for model specifications and the Supplementary Materials for summary statistics.

Robust standard errors clustered at individual level in parentheses.

***p < 0.01, **p < 0.05, *p < 0.1.

This is especially true for the number of days (during the past seven days) respondents report to have stayed at home and not left the house (β = 0.02, *p* < 0.01): a one-unit increase in life satisfaction is associated with an increase in the number of days stayed at home by about 0.2 (about 1% of a standard deviation) in our most restrictive model (i.e. our Arellano-Bond dynamic panel data estimator). The size of the association with this outcome is about the same as anxiety (also about 1% of a standard deviation), but less than confidence in government or the public health service (both about 3%). Associations with other self-reported measures of compliance depended somewhat on the model, but broadly confirm that respondents who are more satisfied with their lives are more likely to show higher levels of compliance behaviour. In Supplementary Materials Table S.2.5, we additionally confirm our previous findings for objective measures of compliance (i.e. from Google Maps) in our longitudinal analysis.

Taking forward our preferred outcome (i.e. the number of weekdays stayed at home), we next use a categorical measure of life satisfaction (Supplementary Materials Table S.2.2). Our Arellano-Bond dynamic panel data estimator suggests that the association between life satisfaction and compliance behaviour is primarily driven by people with above average life satisfaction. The size of the association is strongest for individuals who report a life satisfaction of seven on the zero-to-ten Likert scale (the average is about six) (β = 0.19, *p* < 0.01), or even higher for higher levels. Notably, our first-differences estimator shows that *decreases* in life satisfaction from one week to the next are associated with fewer days stayed at home, and *increases* with more days stayed at home (we discount extreme outliers like individuals changing from zero to ten life satisfaction points).

Finally, we descriptively explore the underlying motivations for compliance with preventive health behaviours, by dividing our sample into two groups: people who are at high risk of severe complications from Covid-19 (defined as being above 60 years of age or having certain medical preconditions, including high blood pressure, diabetes, and heart and lung disease) *versus* people who are at low risk (being below 60 years and having no relevant preconditions). We first look at these two groups during the entire “hard” lockdown period in the UK (Supplementary Materials Table S.2.3). Then, we partition this period into two parts of roughly equal duration, by interacting life satisfaction with a dummy for the pre-April 15 period (Supplementary Materials Table S.2.4). This analysis should be interpreted with caution, as we do not have any questions in our survey data that directly ask respondents about their motivations for compliance. Instead, we infer their motivations from their observed behaviour, and test whether what we observe for life satisfaction aligns with either one of the two dominant psychological theories on how mood and affect influence risk-taking (see Table 1).

Studying the entire “hard” lockdown period in the UK first, we find that associations between life satisfaction and compliance behaviour are strong, significant, and positive for the high-risk group (β = 0.03, *p* < 0.01). For the low-risk group, on the contrary, they are weaker and non-significant (β = 0.01, *p* > 0.1). Looking at the partitioned period, we again find a positive association between life satisfaction and compliance behaviour for the high-risk group (β = 0.02, *p* < 0.05 in our FE model). For the low-risk group, we find that, while life satisfaction is positively associated with compliance during the first half of the “hard” lockdown period in the UK (β = 0.02, *p* < 0.05), this is not the case in the second half (β = − 0.01, *p* > 0.1).

If observed behaviour is reflective of underlying motivations for compliance behaviour, and if predictions from the two predominant psychological theories on how mood and affect influence risk-taking can be transferred to life satisfaction, the observed patterns of compliance between the two groups are in line with the mood maintenance model^21–23^. The strong and positive associations for high-risk individuals consistently throughout both halves of the “hard” lockdown period in the UK may suggest that, when stakes are high, risk-avoidance may be the underlying motivation. For younger and healthier individuals, life satisfaction increases compliance in the first half but not in the second (or even decreases it). This may suggest that, while these individuals may have been motivated by risk-avoidance or pro-sociality at first, their underlying motivation for compliance may have changed as lockdown went on. In fact, only the mood maintenance model predicts a negative association, which may point towards a decrease in pro-sociality over time (see Table 1).

## Discussion

In two independent studies on self-reported compliance with preventive health behaviours from around the world, we find that people’s life satisfaction is predictive of their compliance during Covid-19 lockdowns. Our findings for life satisfaction show that psychological theories on mood and affect could extend to cognitive reflections of how people evaluate their lives. Our study is the first to examine the association of this longer-term measure of subjective wellbeing with preventive health behaviours. In particular, observed patterns of compliance, on average and by risk group, are in line with the mood maintenance model on how mood and affect may influence risk-taking.

**Figure 1:**
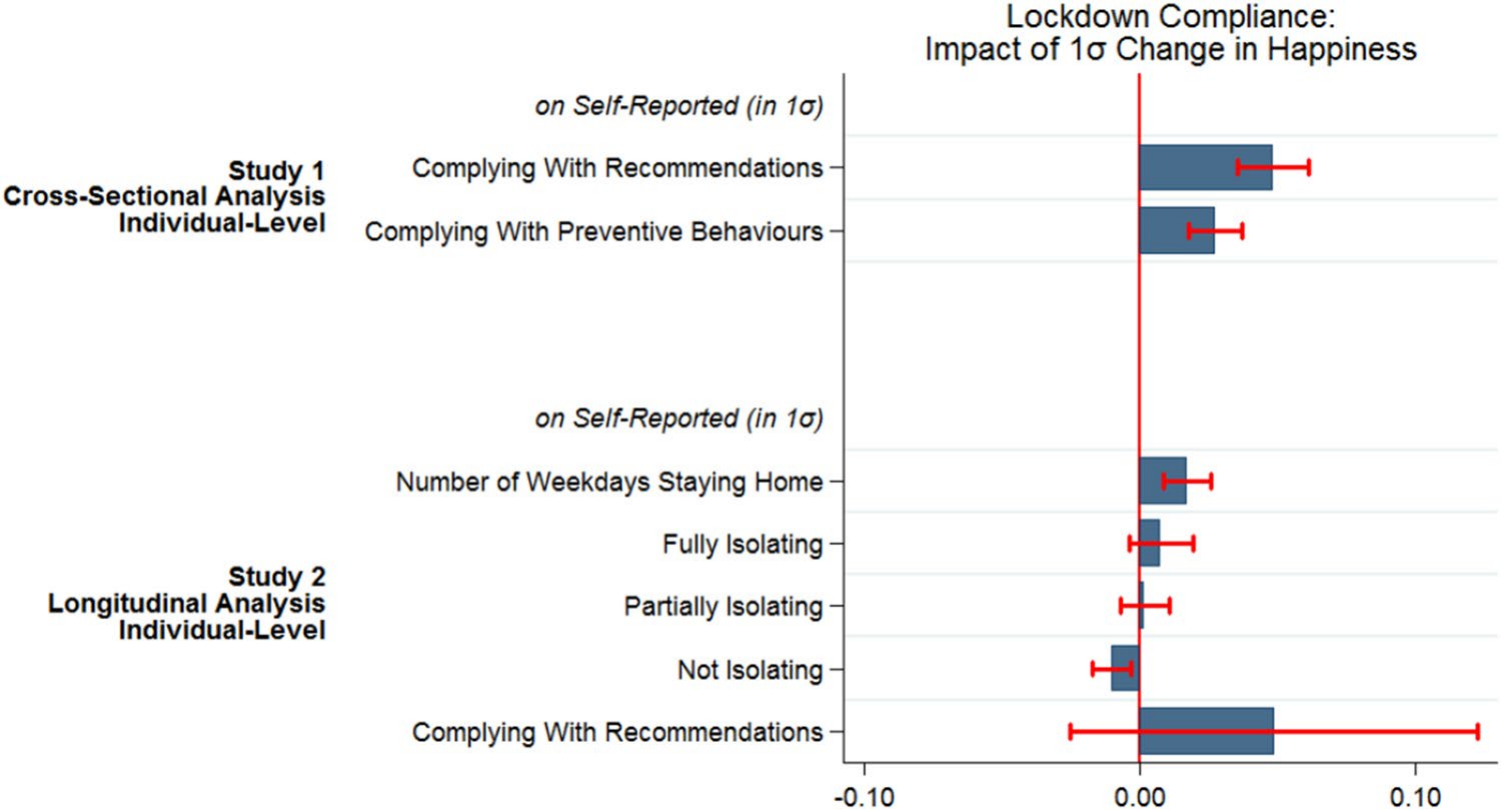
Summary of main findings of studies 1 and 2. *Notes* See Supplementary Materials Table S.1.1 for corresponding table to main findings of Study 1 and main text Table 2 Column 4 for Study 2. See each study in main text for detailed model descriptions. Plotted coefficients for Study 2 come from Arellano-Bond (dynamic panel data) estimator. All coefficients are covariate-adjusted. All variables (i.e. outcomes and variables of interest) were standardised with mean zero and standard deviation one (i.e. z-scores). Confidence bands are 95%.

Figure 1 summarises the main findings from our regression analyses, by showing the association between a one-standard deviation increase in life satisfaction with our main compliance outcomes in Studies 1 and 2, measured in terms of standard deviations.

Our findings hold cross-sectionally between individuals across countries and longitudinally within individuals in a large sample of survey respondents from the UK. For our longitudinal analyses, we use dynamic panel data estimators, holding constant a wide range of time-varying observables, time-invariant unobservables and observables, and, in some models, lagged compliance outcomes. Finally, we find that decreases in life satisfaction are associated with lower compliance, in line with findings on mood by^54^, who show that experimentally inducing negative mood leads to self-defeating behaviours. Associations are modest (about a third of the associations between confidence in government or the public health system and compliance). However, this is not unexpected: our models yield associations for residual life satisfaction, after netting out a wide range of potential confounders.

From our positive association between life satisfaction and compliance with Covid-19 measures, we may cautiously draw some general conclusions about the relationship between compliance behaviour and subjective wellbeing. Goudie et al. (2014) make the argument, rooted in economic theory, that individuals who generate a higher level of utility in their lives (or, in non-economic terms, who enjoy their lives more and hence have more to lose) are more likely to invest in safety-seeking activities and less likely to engage in risk-taking behaviour, e.g. by being non-compliant with government-imposed safety measures^55^. While the debate whether life satisfaction is a valid proxy of utility is out of the scope of our paper, it has been shown that people tend to rank life choices according to how high they score in terms of wellbeing, in particular life satisfaction and happiness^56^. Similar results have been documented by Benjamin et al.^57^, which has led them to suggest that, although people may not maximise wellbeing exclusively, it is ‘a uniquely important argument of the utility function’ (p. 2107). It is therefore likely that our result of a positive association between life satisfaction and compliance with Covid-19 measures may generalise to other forms of compliance with public safety measures, though more evidence on this relationship in other contexts is certainly required. Studies that find an association between positive affect and health behaviours such as medical compliance with treatments for heart failure do point towards this direction^58,59^.

There are several limitations to this paper. Although we employ two large-scale cross-sectional and longitudinal surveys covering a wide range of countries, each of them is likely to underrepresent the most reluctant non-compliers (who are unlikely to participate in surveys in the first place). Due to the observational nature of our study, and the constructs examined (i.e. overall life satisfaction), we are unable to make causal inferences. The experimental manipulation of life satisfaction (as a long-term construct) needed to formally claim causality is difficult on both logistical and ethical grounds. However, our use of multiple datasets, multiple measures of compliance behaviour, and our global sample help to buffer against spurious correlational claims.

As a word of caution, it should be noted that it is difficult to estimate the relationship between life satisfaction and compliance behaviour, due to potential confounders and unobserved heterogeneity that are difficult to control for without exogenous variation in life satisfaction. The fact that our estimates become smaller and less significant as our models become more restrictive are testimony to this. As such, our finding of a significant relationship in our most restrictive model (amongst the many compliance behaviours that have been investigated) should be taken with caution.

A potential issue is the reliability of self-reports of subjective wellbeing in different regions of the Likert scale, and in particular, whether individuals who report a higher life satisfaction may be more likely to misreport their compliance behaviour, for whatever reason. This may be particularly problematic when *overstating* compliance, as this would yield an upper-bound association (potentially including a null association). While this concern is difficult to conclusively disprove, there is evidence that happier individuals report their behaviour, if anything, *more truthfully*. In a recent paper, it has been show that experimentally inducing incidental happiness by means of video clips leads to more truthful reporting in the die-rolling task by Fischbacher and Föllmi-Heusi, a standard experimental paradigm to test for dishonesty and lying, relative to a neutral control condition^60,61^. Acknowledging that experiences of happiness are conceptually different (though related) to global life evaluations, this suggests that individuals who report a higher life satisfaction are more, not less, likely to truthfully report their behaviour. Note that life satisfaction is a standard measure in the literature that has been thoroughly scrutinised regarding its validity and reliability, and is now routinely used for policy appraisal and evaluation, for example in the UK^62,63^. To our knowledge, no framing or priming was involved in any of our survey instruments, implying that the relationship between compliance behaviour and life satisfaction was never openly visible to respondents at any point in time, and neither was the purpose of our study, thereby reducing incentives to respond in a strategical or socially desirable manner.

An important issue throughout our regressions is to control for income, which is a potential confounder that may be positively correlated with both compliance behaviour and life satisfaction. Omitting income would, therefore, yield omitted variable bias, and hence an overestimate of the true association between compliance behaviour and life satisfaction. While we are able to control for household income (measured in income brackets) in Study 2, we lack a variable on income in Study 1, which is based on the Imperial College London-YouGov Covid-19 Behavioural Tracker and which does not ask respondents about their incomes. Acknowledging this limitation, we are trying to mitigate it by controlling for country fixed effects (i.e. differences in income levels at the country level), using a short observation period (i.e. about two months in each country, over which income levels should be relatively stable), and restricting our sample to individuals who are subject to a homogeneous policy response to Covid-19 (which may limit the choices people have as a function of their incomes).

Finally, our data are not detailed enough to identify the specific mechanisms by which life satisfaction may raise compliance behaviour, though we do provide some suggestive evidence. The observed patterns of compliance behaviour between individuals with high-risk and low-risk of severe complications from Covid-19 are in line with predictions made by the mood maintenance model of mood and risk-taking, as opposed to the affect infusion model. However, underlying motivations seem to be complex and probably highly context-dependent: the same person may be motivated by risk-avoidance in one context at one point in time and pro-socially in another at another point in time. It is difficult to differentiate these empirically from observed behaviour alone. While our data are not granular enough to disentangle them, future research may explore in more depth the underlying mechanisms between life satisfaction and compliance behaviours.

Governments and public health officials often rely on people’s voluntary compliance with preventive health behaviours. Our work shows that people’s subjective evaluations of their lives are predictive of their compliance with such measures. And, while our research does not allow strictly causal inferences, it provides important guidance for public health officials when weighing future policy options. We show that a focus on maintaining wellbeing can improve compliance with collective health policies, as well be an aim in itself. What is more, policies aimed at improving wellbeing, either directly or indirectly, may prove to be particularly cost-effective and worthwhile, by having positive spillovers on various unrelated life domains, including productivity^1,2^, longevity^3,4^, and a wide range of other positive behaviours^11^. As wellbeing has also been shown to predict voting for the incumbent government^7–9^, policies aimed at improving wellbeing should therefore be in the self-inherent interest of policy-makers.

## Supplementary Information

Supplementary Information.

## Data Availability

All relevant raw data and scripts, including for the creation of the estimation samples and for the generation of our findings, will be made freely available to any researcher wishing to use them for non-commercial purposes, without breaching participant confidentiality. These will be uploaded on PsyArXiv, in our project folder.
