# Supplementary material for: Happiness predicts compliance with preventive health behaviours during Covid-19 lockdowns
Source: Sci Rep. 2023 May 17;13:7989. doi: 10.1038/s41598-023-33136-9 (PMC10189679; doi:10.1038/s41598-023-33136-9)
Supplement: Supplementary file 1 — Supplementary Information. [file 41598_2023_33136_MOESM1_ESM.docx]

**Supplementary Materials**

***Study 1: Cross-Sectional Evidence on Individual Life Satisfaction and Self-Reported Individual Compliance***

***Methods***

We regress self-reported compliance behaviour on respondents’ present life satisfaction during Covid-19 lockdowns:

*compliance_i_ = a + β_1_satisfaction_i_ + X_i_β_1_' + η_wd_ + η_w_ + μ_c_ + ε_i_* (2)

where *compliance_i_* is the compliance behaviour of individual *i*, *satisfaction_i_* is the life satisfaction of individual *i*, *X* is a vector of controls, and *η_wd_* are weekday, *η_w_* calendar week, and *μ_c_* country fixed effects. The controls include age, gender, employment status, and the number of adults and children in their household, as well as the log daily numbers of confirmed Covid-19 cases and deaths at the country level. The model is estimated using OLS, with robust standard errors clustered at the interview date level.

Our analysis includes the following countries and observation periods: Australia (1 April to 11 June, 2020), Brazil (2 April to 14 June), Canada (2 April to 22 June), China (2 April to 14 June), Denmark (9 April to 11 June), Finland (8 April to 15 June), France (2 April to 11 June), Germany (2 April to 12 June), Hong Kong (9 April to 22 June), India (1 April to 12 June), Indonesia (8 April to 11 June), Italy (2 April to 12 June), Japan (2 April to 19 June), Malaysia (9 April to 11 June), Mexico (3 April to 13 June), Netherlands (9 April to 28 May), Norway (9 April to 14 June), Philippines (8 April to 11 June), Saudi Arabia (8 April to 21 June), Singapore (3 April to 12 June), South Korea (2 April to 21 June), Spain (2 April to 14 June), Sweden (3 April to 13 June), Taiwan (8 April to 11 June), Thailand (9 April to 11 June), United Arab Emirates (9 April to 19 June), United Kingdom (1 April to 18 June), United States (2 April to 17 June), and Vietnam (9 April to 11 June).

| *Table S.1.1: Life Satisfaction and Self-Reported Compliance (Imperial College London-YouGov Covid-19 Behaviour Tracker, 29 Countries, Year 2020)* | | | | |
| --- | --- | --- | --- | --- |
|  |  |  |  |  |
|  | | Complying  With Recommendations | Complying With Preventive Health Behaviours (Index) |  |
|  | | (1) | (2) |  |
|  | |  |  |  |
| Life Satisfaction | | 0.0083*** | 0.0133*** |  |
|  | | (0.0011) | (0.0023) |  |
|  | |  |  |  |
| Controls | | Yes | Yes |  |
| Country Fixed Effects | | Yes | Yes |  |
| Week-Day Fixed Effects | | Yes | Yes |  |
| Week Fixed Effects | | Yes | Yes |  |
|  | |  |  |  |
| Observations | | 38,910 | 12,520 |  |
| R² | | 0.025 | 0.148 |  |
| See Study 1 in main text for model specifications.  Robust standard errors clustered at daily date level in parentheses.  *** p<0.01, ** p<0.05, * p<0.1 | | | |  |

| *Table S.1.2: Life Satisfaction and Self-Reported Compliance, All Preventive Behaviours (Imperial College London-YouGov Covid-19 Behaviour Tracker, 29 Countries, Year 2020)* | | | | | |
| --- | --- | --- | --- | --- | --- |
|  | Wearing Face Mask | Washing Hands | Using Sanitiser | Covering Mouth, Nose | Avoiding Contact |
|  | (1) | (2) | (3) | (4) | (5) |
| Life Satisfaction | 0.0016 | 0.0037*** | 0.0097*** | 0.0043*** | 0.0049*** |
|  | (0.0010) | (0.0007 | (0.0014) | (0.0007) | (0.0012) |
|  |  |  |  |  |  |
| Observations | 39,837 | 39,837 | 39,837 | 39,837 | 39,837 |
| R² | 0.352 | 0.021 | 0.088 | 0.031 | 0.038 |
|  |  |  |  |  |  |
|  | Staying In | Avoiding Healthcare | Avoiding Transit | Doing Home Office | Avoiding Schooling |
|  | (6) | (7) | (8) | (9) | (10) |
| Life Satisfaction | -0.0012 | -0.0004 | 0.0040*** | 0.0084*** | 0.0017 |
|  | (0.0016 | (0.0014) | (0.0012) | (0.0019) | (0.0011) |
|  |  |  |  |  |  |
| Observations | 39,837 | 39,837 | 39,837 | 23,216 | 18,911 |
| R² | 0.077 | 0.045 | 0.065 | 0.065 | 0.094 |
|  |  |  |  |  |  |
|  | Avoiding Guests | Avoiding  Small Gatherings | Avoiding  Medium Gatherings | Avoiding  Large Gatherings | Avoiding  Crowded Areas |
|  | (11) | (12) | (13) | (14) | (15) |
| Life Satisfaction | -0.0004 | 0.0001 | 0.0001 | 0.0006 | 0.0028** |
|  | (0.0010) | (0.0014) | (0.0009) | (0.0009) | (0.0011) |
|  |  |  |  |  |  |
| Observations | 39,837 | 39,837 | 39,837 | 39,837 | 39,837 |
| R² | 0.079 | 0.060 | 0.050 | 0.039 | 0.034 |
|  |  |  |  |  |  |
|  | Avoiding Shopping | Sleeping Separately | Eating Separately | Cleaning More Often | Avoiding  Touching Objects |
|  | (16) | (17) | (18) | (19) | (20) |
| Life Satisfaction | 0.0037** | -0.0046** | -0.0020 | 0.0092*** | 0.0085*** |
|  | (0.0014) | (0.0022) | (0.0015) | (0.0018) | (0.0013) |
|  |  |  |  |  |  |
| Observations | 39,837 | 39,837 | 39,837 | 39,837 | 39,837 |
| R² | 0.080 | 0.073 | 0.062 | 0.080 | 0.049 |
|  |  |  |  |  |  |
| Controls | Yes | Yes | Yes | Yes | Yes |
| Country Fixed Effects | Yes | Yes | Yes | Yes | Yes |
| Week-Day Fixed Effects | Yes | Yes | Yes | Yes | Yes |
| Week Fixed Effects | Yes | Yes | Yes | Yes | Yes |
| See Study 1 in main text for model specifications.  Robust standard errors clustered at daily date level in parentheses. | | | | | |
| *** p<0.01, ** p<0.05, * p<0.1 | | | | | |

| *Table S.1.3: Life Satisfaction and Self-Reported Compliance (Imperial College London-YouGov Covid-19 Behaviour Tracker, 29 Countries, Year 2020)  – No Stringency or Person Restrictions* | | | | |
| --- | --- | --- | --- | --- |
|  |  |  |  |  |
|  | | Complying  With Recommendations | Complying With Preventive Health Behaviours (Index) |  |
|  | | (1) | (2) |  |
|  | |  |  |  |
| Life Satisfaction | | 0.0087*** | 0.0176*** |  |
|  | | (0.0007) | (0.0019) |  |
|  | |  |  |  |
| Controls | | Yes | Yes |  |
| Country Fixed Effects | | Yes | Yes |  |
| Week-Day Fixed Effects | | Yes | Yes |  |
| Week Fixed Effects | | Yes | Yes |  |
|  | |  |  |  |
| Observations | | 86,943 | 27,800 |  |
| R² | | 0.039 | 0.129 |  |
| See Study 1 in main text for model specifications.  Robust standard errors clustered at daily date level in parentheses.  *** p<0.01, ** p<0.05, * p<0.1 | | | |  |

| *Table S.1.4: Life Satisfaction and Self-Reported Compliance, All Preventive Behaviours (Imperial College London-YouGov Covid-19 Behaviour Tracker, 29 Countries, Year 2020) – No Stringency or Person Restrictions* | | | | | |
| --- | --- | --- | --- | --- | --- |
|  | Wearing Face Mask | Washing Hands | Using Sanitiser | Covering Mouth, Nose | Avoiding Contact |
|  | (1) | (2) | (3) | (4) | (5) |
| Life Satisfaction | 0.0042*** | 0.0045*** | 0.0090*** | 0.0051*** | 0.0055*** |
|  | (0.0008) | (0.0005) | (0.0009) | (0.0007) | (0.0009) |
|  |  |  |  |  |  |
| Observations | 89,401 | 89,397 | 89,406 | 89,407 | 89,395 |
| R² | 0.366 | 0.032 | 0.088 | 0.033 | 0.040 |
|  |  |  |  |  |  |
|  | Staying In | Avoiding Healthcare | Avoiding Transit | Doing Home Office | Avoiding Schooling |
|  | (6) | (7) | (8) | (9) | (10) |
| Life Satisfaction | 0.0009 | 0.0028*** | 0.0062*** | 0.0104*** | 0.0053*** |
|  | (0.0011) | (0.0008) | (0.0008) | (0.0010) | (0.0011) |
|  |  |  |  |  |  |
| Observations | 89,398 | 89,398 | 89,396 | 53,056 | 40,916 |
| R² | 0.080 | 0.048 | 0.092 | 0.077 | 0.105 |
|  |  |  |  |  |  |
|  | Avoiding Guests | Avoiding  Small Gatherings | Avoiding  Medium Gatherings | Avoiding  Large Gatherings | Avoiding  Crowded Areas |
|  | (11) | (12) | (13) | (14) | (15) |
| Life Satisfaction | 0.0022*** | 0.0018* | 0.0019** | 0.0020*** | 0.0046*** |
|  | (0.0007) | (0.0010) | (0.0008) | (0.0007) | (0.0010) |
|  |  |  |  |  |  |
| Observations | 89,400 | 89,399 | 89,401 | 89,399 | 89,400 |
| R² | 0.090 | 0.074 | 0.064 | 0.050 | 0.048 |
|  |  |  |  |  |  |
|  | Avoiding Shopping | Sleeping Separately | Eating Separately | Cleaning More Often | Avoiding  Touching Objects |
|  | (16) | (17) | (18) | (19) | (20) |
| Life Satisfaction | 0.0052*** | -0.0028** | 0.0002 | 0.0098*** | 0.0096*** |
|  | (0.0011) | (0.0014) | (0.0011) | (0.0010) | (0.0009) |
|  |  |  |  |  |  |
| Observations | 89,407 | 89,399 | 89,406 | 89,400 | 89,400 |
| R² | 0.090 | 0.073 | 0.062 | 0.077 | 0.052 |
|  |  |  |  |  |  |
| Controls | Yes | Yes | Yes | Yes | Yes |
| Country Fixed Effects | Yes | Yes | Yes | Yes | Yes |
| Week-Day Fixed Effects | Yes | Yes | Yes | Yes | Yes |
| Week Fixed Effects | Yes | Yes | Yes | Yes | Yes |
| See Study 1 in main text for model specifications.  Robust standard errors clustered at daily date level in parentheses. | | | | | |
| *** p<0.01, ** p<0.05, * p<0.1 | | | | | |

| *Table S.1.5: Summary Statistics (Imperial College London-YouGov Covid-19 Behaviour Tracker, 29 Countries, Year 2020)* | | | | | |
| --- | --- | --- | --- | --- | --- |
| Variable | Mean | Standard Deviation | Minimum | Maximum | Number of Observations |
|  |  |  |  |  |  |
| *Self-Reported Outcomes* |  |  |  |  |  |
| Complying With Recommendations | 0.8680 | 0.3385 | 0 | 1 | 38,910 |
| Complying With Preventive Health Behaviours (Index) | 0.1797 | 0.4372 | -1.6253 | 0.7752 | 12,244 |
| Wearing Face Mask | 0.7737 | 0.4185 | 0 | 1 | 38,910 |
| Washing Hands | 0.9541 | 0.2093 | 0 | 1 | 38,910 |
| Using Sanitiser | 0.7685 | 0.4218 | 0 | 1 | 38,910 |
| Covering Mouth, Nose | 0.9291 | 0.2567 | 0 | 1 | 38,910 |
| Avoiding Contact | 0.8878 | 0.3156 | 0 | 1 | 38,910 |
| Staying In | 0.7685 | 0.4218 | 0 | 1 | 38,910 |
| Avoiding Healthcare | 0.8120 | 0.3907 | 0 | 1 | 38,910 |
| Avoiding Transit | 0.8529 | 0.3542 | 0 | 1 | 38,910 |
| Doing Home Office | 0.6564 | 0.4749 | 0 | 1 | 22,760 |
| Avoiding Schooling | 0.8338 | 0.3722 | 0 | 1 | 18,393 |
| Avoiding Guests | 0.8539 | 0.3532 | 0 | 1 | 38,910 |
| Avoiding Small Gatherings | 0.8170 | 0.3867 | 0 | 1 | 38,910 |
| Avoiding Medium Gatherings | 0.8790 | 0.3262 | 0 | 1 | 38,910 |
| Avoiding Large Gatherings | 0.9203 | 0.2708 | 0 | 1 | 38,910 |
| Avoiding Crowded Areas | 0.9116 | 0.2839 | 0 | 1 | 38,910 |
| Avoiding Shopping | 0.6750 | 0.4684 | 0 | 1 | 38,910 |
| Sleeping Separately | 0.3425 | 0.4745 | 0 | 1 | 38,910 |
| Eating Separately | 0.3442 | 0.4751 | 0 | 1 | 38,910 |
| Cleaning More Often | 0.6894 | 0.4628 | 0 | 1 | 38,910 |
| Avoiding Touching Objects | 0.8095 | 0.3927 | 0 | 1 | 38,910 |
|  |  |  |  |  |  |
| *Variables of Interest* |  |  |  |  |  |
| Life Satisfaction | 6.3340 | 1.9230 | 0 | 10 | 38,910 |
|  |  |  |  |  |  |
| *Controls* |  |  |  |  |  |
| Age | 44.0376 | 15.7984 | 18 | 91 | 38,910 |
| Female | 0.5178 | 0.4997 | 0 | 1 | 38,910 |
| Full-Time Employed | 0.4580 | 0.4982 | 0 | 1 | 38,910 |
| Part-Time Employed | 0.1315 | 0.3379 | 0 | 1 | 38,910 |
| Unemployed | 0.0969 | 0.2959 | 0 | 1 | 38,910 |
| Not Working | 0.0807 | 0.2724 | 0 | 1 | 38,910 |
| Retired | 0.1391 | 0.3460 | 0 | 1 | 38,910 |
| Other Employment | 0.0244 | 0.1542 | 0 | 1 | 38,910 |
| Number of Children in Household | 3.1893 | 1.4869 | 1 | 8 | 38,910 |
| Number of Individuals in Household | 0.9158 | 1.2307 | 0 | 5 | 38,910 |
| Number of New Covid-19 Cases | 240,086.6 | 421,218.6 | 1,112 | 2,074,526 | 38,910 |
| Number of New Covid-19 Deaths | 19,008.5 | 25,650.34 | 4 | 115,436 | 38,910 |

***Study 2: Longitudinal Evidence on Individual Life Satisfaction and Self-Reported Individual Compliance***

***Methods***

We estimate the following models:

*compliance_it_ = a + β_1_satisfaction_it_ + X_it_β_2_' + η_wd_ + μ_c_ + ε_it_* (2.1)

*compliance_it_ = a + β_1_satisfaction_it_ + X_it_β_2_' + η_wd_ + μ_c_ + μ_i_ + ε_it_* (2.2)

*compliance_it_ = a + β_1_satisfaction_it_ + X_it_β_2_' + β_3_compliance_it-1_ + η_wd_ + μ_c_ + μ_i_ + ε_it_* (2.3)

where *compliance_it_* is the compliance behaviour of individual *i* in week *t*, *satisfaction_it_* is the life satisfaction of individual *i* in week *t*, *X_it_* is a vector of controls, and *η_wd_* are weekday, *μ_c_* country, and *μ_i_* individual fixed effects. The controls include age, gender, marital status, ethnicity, education, employment status, income, dwelling type, the number of rooms in the dwelling, the number of children and adults in the household, the residential area (city, large town, small town, village, hamlet, or isolated dwelling), the number of close friends, the frequency of socialising (in normal times), self-assessed knowledge about Covid-19, confidence in government, confidence in the public health system, the Big-5 personality traits, measures of depression (PHQ-9) and anxiety (GAD-7), as well as the log numbers of both daily confirmed Covid-19 cases and daily confirmed Covid-19 deaths at the country level. Robust standard errors are clustered at the individual level (clustering at the interview date level yields similar results).

| *Table S.2.1: Life Satisfaction and Self-Reported Compliance, With Week Fixed Effects (University College London Covid-19 Social Study, UK, Year 2020)* | | | | |
| --- | --- | --- | --- | --- |
|  | Static Panel Data Estimation | | | Dynamic Panel Data Estimation |
|  | Pooled OLS | FE | FD | Arellano-Bond |
|  | (1) | (2) | (3) | (4) |
| *Panel A: Number of Weekdays Staying Home* | | | | |
| Life Satisfaction | 0.0550*** | 0.0024 | 0.0154*** | 0.0157*** |
|  | (0.0064) | (0.0055) | (0.0059) | (0.0051) |
|  |  |  |  |  |
| Hansen Test P-Value | - | - | - | n.s. |
| AR(1), AR(2), AR(3) P-Values | - | - | - | 0.000, 0.000, 0.235 |
| Observations | 131,088 | 131,088 | 99,403 | 100,850 |
| Individuals | 34,136 | 34,136 | 28,897 | 29,753 |
| R² (GMM = F) | 0.094 | 0.025 | 0.014 | F(86, 29752) = 5464.18 |
|  |  |  |  |  |
| *Panel B: Fully Isolating* | | | | |
| Life Satisfaction | 0.0012* | -0.0015** | -0.0002 | 0.0009* |
|  | (0.0007) | (0.0007) | (0.0006) | (0.0005) |
|  |  |  |  |  |
| Hansen Test P-Value | - | - | - | n.s. |
| AR(1), AR(2), AR(3) P-Values | - | - | - | 0.000, 0.010, 0.764 |
| Observations | 132,703 | 132,703 | 102,327 | 104,020 |
| Individuals | 34,046 | 34,046 | 29,040 | 29,967 |
| R² (GMM = F) | 0.100 | 0.084 | 0.033 | F(86, 29966) = 178.88 |
|  |  |  |  |  |
| *Panel C: Partially Isolating* | | | | |
| Life Satisfaction | 0.0007 | 0.0026*** | 0.0007 | -0.0007 |
|  | (0.0007) | (0.0010) | (0.0008) | (0.0008) |
|  |  |  |  |  |
| Hansen Test P-Value | - | - | - | n.s. |
| AR(1), AR(2), AR(3) P-Values | - | - | - | 0.000, 0.000, 0.223 |
| Observations | 132,703 | 132,703 | 102,327 | 104,020 |
| Individuals | 34,046 | 34,046 | 29,040 | 29,967 |
| R² (GMM = F) | 0.064 | 0.036 | 0.029 | F(86, 29966) = 108.88 |
|  |  |  |  |  |
| *Panel D: Not Isolating* | | | | |
| Life Satisfaction | -0.0018*** | -0.0012* | -0.0005 | -0.0007 |
|  | (0.0004) | (0.0007) | (0.0006) | (0.0011) |
|  |  |  |  |  |
| Hansen Test P-Value | - | - | - | n.s. |
| AR(1), AR(2), AR(3) P-Values | - | - | - | 0.503, 0.552, 0.703 |
| Observations | 132,703 | 132,703 | 102,327 | 104,020 |
| Individuals | 34,046 | 34,046 | 29,040 | 29,967 |
| R² (GMM = F) | 0.149 | 0.167 | 0.041 | F(86, 29966) = 60.71 |
|  |  |  |  |  |
| *Panel E: Complying With Recommendations* | | | | |
| Life Satisfaction | 0.0143*** | 0.0010 | 0.0003 | 0.0080 |
|  | (0.0015) | (0.0013) | (0.0015) | (0.0078) |
|  |  |  |  |  |
| Hansen Test P-Value | - | - | - | n.s. |
| AR(1), AR(2), AR(3) P-Values | - | - | - | 0.306, 0.642, 0.733 |
| Observations | 136,385 | 136,385 | 107,278 | 108,298 |
| Individuals | 34,378 | 34,378 | 29,548 | 29,713 |
| R² (GMM = F) | 0.108 | 0.020 | 0.022 | F(86, 29712) = 78.95 |
|  |  |  |  |  |
| Controls | Yes | Yes | Yes | Yes |
| Area Fixed Effects | Yes | Yes | Yes | Yes |
| Country Fixed Effects | Yes | Yes | Yes | Yes |
| Week-Day Fixed Effects | Yes | Yes | Yes | Yes |
| Week Fixed Effects | Yes | Yes | Yes | Yes |
| Individual Fixed Effects | No | Yes | Yes | Yes |
| See Study 2 in main text for model specifications.  Robust standard errors clustered at individual level in parentheses.  *** p<0.01, ** p<0.05, * p<0.1 | | | | |

| *Table S.2.2: Life Satisfaction and Self-Reported Compliance (University College London Covid-19 Social Study, UK, Year 2020), By Wellbeing* | | | | |
| --- | --- | --- | --- | --- |
|  | Static Panel Data Estimation | | | Dynamic Panel Data Estimation |
|  | Pooled OLS | FE | FD | Arellano-Bond |
|  | (1) | (2) | (3) | (4) |
| *Number of Weekdays Staying Home* | | | | |
| Life Satisfaction = 0 (FD = +0) | *Reference Category* |  |  |  |
|  |  |  |  |  |
| = 1 (FD: Δ = +1) | 0.2210** | 0.0514 | 0.0216 | 0.0939 |
|  | (0.0939) | (0.0722) | (0.0195) | (0.0836) |
| = 2 (FD: Δ = +2) | 0.2990*** | 0.0059 | 0.0273 | 0.0248 |
|  | (0.0891) | (0.0732) | (0.0308) | (0.0722) |
| = 3 (FD: Δ = +3) | 0.2910*** | 0.0410 | 0.1150** | 0.1200* |
|  | (0.0872) | (0.0729) | (0.0499) | (0.0684) |
| = 4 (FD: Δ = +4) | 0.3860*** | 0.0667 | -0.0753 | 0.1490** |
|  | (0.0876) | (0.0743) | (0.0847) | (0.0687) |
| = 5 (FD: Δ = +5) | 0.4560*** | 0.0904 | 0.1920 | 0.1240* |
|  | (0.0868) | (0.0741) | (0.1400) | (0.0677) |
| = 6 (FD: Δ = +6) | 0.4300*** | 0.0942 | -0.1430 | 0.1310* |
|  | (0.0875) | (0.0750) | (0.2790) | (0.0683) |
| = 7 (FD: Δ = +7) | 0.5270*** | 0.1310* | 0.5800 | 0.1990*** |
|  | (0.0879) | (0.0754) | (0.5530) | (0.0690) |
| = 8 (FD: Δ = +8) | 0.5900*** | 0.1480* | -0.4900 | 0.2120*** |
|  | (0.0899) | (0.0767) | (0.9030) | (0.0708) |
| = 9 (FD: Δ = +9) | 0.6960*** | 0.1420* | 0.5850 | 0.1580** |
|  | (0.0959) | (0.0814) | (4.5040) | (0.0760) |
| = 10 (FD: Δ = +10) | 0.8440*** | 0.1500 | -0.1830*** | 0.2790*** |
|  | -0.108 | (0.0938) | (0.0263) | (0.0855) |
| = Δ = -1 |  |  | -0.0259 |  |
|  |  |  | (0.0194) |  |
| = Δ = -2 |  |  | -0.0279 |  |
|  |  |  | (0.0295) |  |
| = Δ = -3 |  |  | -0.0486 |  |
|  |  |  | (0.0461) |  |
| = Δ = -4 |  |  | -0.1440* |  |
|  |  |  | (0.0798) |  |
| = Δ = -5 |  |  | -0.3110** |  |
|  |  |  | (0.1390) |  |
| = Δ = -6 |  |  | 0.1230 |  |
|  |  |  | (0.2850) |  |
| = Δ = -7 |  |  | -0.8820 |  |
|  |  |  | (0.7890) |  |
| = Δ = -8 |  |  | -1.1240 |  |
|  |  |  | (0.8260) |  |
| = Δ = -9 |  |  | 1.7710 |  |
|  |  |  | (1.7090) |  |
| = Δ = -10 |  |  |  |  |
|  |  |  |  |  |
|  |  |  |  |  |
| Hansen Test P-Value | - | - | - | 0.759 |
| AR(1), AR(2), AR(3) P-Values | - | - | - | 0.000, 0.000, 0.109 |
| Observations | 131,088 | 131,088 | 99,403 | 100,850 |
| Individuals | 34,136 | 34,136 | 28,897 | 29,753 |
| R² (GMM = F) | 0.086 | 0.006 | 0.005 | F(86, 29752) = 230.86 |
|  |  |  |  |  |
| Controls | Yes | Yes | Yes | Yes |
| Area Fixed Effects | Yes | Yes | Yes | Yes |
| Country Fixed Effects | Yes | Yes | Yes | Yes |
| Week-Day Fixed Effects | Yes | Yes | Yes | Yes |
| Week Fixed Effects | No | No | No | No |
| Individual Fixed Effects | No | Yes | Yes | Yes |
| See Study 2 in main text for model specifications.  Robust standard errors clustered at individual level in parentheses.  *** p<0.01, ** p<0.05, * p<0.1 | | | | |

| *Table S.2.3: Life Satisfaction and Self-Reported Compliance (University College London Covid-19 Social Study, UK, Year 2020), By Risk* | | | | |
| --- | --- | --- | --- | --- |
|  | Number of Weekdays Staying Home | | | |
|  | Static Panel Data Estimation | | | Dynamic Panel Data Estimation |
|  | Pooled OLS | FE | FD | Arellano-Bond |
|  | (1) | (2) | (3) | (4) |
| *Panel A: High Risk (≥60 Years or Medical Preconditions)* | | | | |
| Life Satisfaction | 0.0634*** | 0.0291*** | 0.0339*** | 0.0302*** |
|  | (0.0090) | (0.0078) | (0.0084) | (0.00707) |
|  |  |  |  |  |
| Hansen Test P-Value | - | - | - | 0.136 |
| AR(1), AR(2), AR(3) P-Values | - | - | - | 0.000, 0.000, 0.039 |
| Observations | 71,116 | 71,116 | 53,526 | 54,345 |
| Individuals | 17,795 | 17,795 | 15,391 | 15,848 |
| R² (GMM = F) | 0.092 | 0.010 | 0.006 | F(77, 15847) = 145.66 |
|  |  |  |  |  |
| *Panel B: Low Risk (<60 Years and No Medical Preconditions)* | | | | |
| Life Satisfaction | 0.0613*** | 0.0053 | 0.0065 | 0.00853 |
|  | (0.0089) | (0.0079) | (0.0084) | (0.00730) |
|  |  |  |  |  |
| Hansen Test P-Value | - | - | - | 0.172 |
| AR(1), AR(2), AR(3) P-Values | - | - | - | 0.000, 0.000, 0.919 |
| Observations | 59,972 | 59,972 | 45,877 | 46,505 |
| Individuals | 16,341 | 16,341 | 13,506 | 13,905 |
| R² (GMM = F) | 0.082 | 0.005 | 0.005 | F(77, 13904) = 123.62 |
|  |  |  |  |  |
| Controls | Yes | Yes | Yes | Yes |
| Area Fixed Effects | Yes | Yes | Yes | Yes |
| Country Fixed Effects | Yes | Yes | Yes | Yes |
| Week-Day Fixed Effects | Yes | Yes | Yes | Yes |
| Week Fixed Effects | No | No | No | No |
| Individual Fixed Effects | No | Yes | Yes | Yes |
| See Study 2 for model specifications.  Robust standard errors clustered at individual level in parentheses.  *** p<0.01, ** p<0.05, * p<0.1 | | | | |

| *Table S.2.4: Life Satisfaction and Self-Reported Compliance (University College London Covid-19 Social Study, UK, Year 2020), By Risk Over Time* | | | | |
| --- | --- | --- | --- | --- |
|  | Number of Weekdays Staying Home | | | |
|  | Static Panel Data Estimation | | | Dynamic Panel Data Estimation |
|  | Pooled OLS | FE | FD | Arellano-Bond |
|  | (1) | (2) | (3) | (4) |
| *Panel A: High Risk (≥60 Years or Medical Preconditions)* | | | | |
| Life Satisfaction * Pre April 15 | -0.0058 | 0.0008 | -0.0089 | 0.2430 |
|  | (0.0089) | (0.0075) | (0.0168) | (0.2270) |
| Life Satisfaction | 0.0598*** | 0.0169** | 0.0374*** | -0.0328 |
|  | (0.0097) | (0.0085) | (0.0107) | (0.0745) |
| Pre April 15 | 0.5580*** | 0.4240*** | -0.0105 | -2.7880 |
|  | (0.0590) | (0.0495) | (0.0392) | (2.2760) |
|  |  |  |  |  |
| Hansen Test P-Value | - | - | - | 0.011 |
| AR(1), AR(2), AR(3) P-Values | - | - | - | 0.000, 0.001, 0.088 |
| Observations | 71,116 | 71,116 | 53,526 | 54,345 |
| Individuals | 17,795 | 17,795 | 15,391 | 15,848 |
| R² (GMM = F) | 0.099 | 0.021 | 0.006 | F(79, 15847) = 78.37 |
|  |  |  |  |  |
| *Panel B: Low Risk (<60 Years and No Medical Preconditions)* | | | | |
| Life Satisfaction * Pre April 15 | -0.0089 | 0.0195** | 0.0252 | 0.2510 |
|  | (0.0095) | (0.0083) | (0.0166) | (0.1980) |
| Life Satisfaction | 0.0621*** | -0.0094 | -0.0045 | -0.0876 |
|  | (0.0100) | (0.0087) | (0.0099) | (0.0827) |
| Pre April 15 | 0.3650*** | 0.1150** | 0.0480 | -1.5250 |
|  | (0.0594) | (0.0519) | (0.0391) | (1.3870) |
|  |  |  |  |  |
| Hansen Test P-Value | - | - | - | 0.551 |
| AR(1), AR(2), AR(3) P-Values | - | - | - | 0.000, 0.000, 0.930 |
| Observations | 59,972 | 59,972 | 45,877 | 46,505 |
| Individuals | 16,341 | 16,341 | 13,506 | 13,905 |
| R² (GMM = F) | 0.085 | 0.008 | 0.005 | F(79, 13904) = 115.95 |
|  |  |  |  |  |
| Controls | Yes | Yes | Yes | Yes |
| Area Fixed Effects | Yes | Yes | Yes | Yes |
| Country Fixed Effects | Yes | Yes | Yes | Yes |
| Week-Day Fixed Effects | Yes | Yes | Yes | Yes |
| Week Fixed Effects | No | No | No | No |
| Individual Fixed Effects | No | Yes | Yes | Yes |
| See Study 2 in main text for model specifications.  Robust standard errors clustered at individual level in parentheses.  *** p<0.01, ** p<0.05, * p<0.1 | | | | |

| *Table S.2.5: Life Satisfaction and Observed Compliance (University College London Covid-19 Social Study, UK, Year 2020)* | | | | |
| --- | --- | --- | --- | --- |
|  | Static Panel Data Estimation | | | Dynamic Panel Data Estimation |
|  | Pooled OLS | FE | FD | Arellano-Bond |
|  | (1) | (2) | (3) | (4) |
| *Panel A: Percentage Change in Mobility in Residential Areas* | | | | |
| Life Satisfaction | 0.0284*** | 0.0811*** | 0.0714*** | 0.0265** |
|  | (0.0064) | (0.0131) | (0.0173) | (0.0109) |
|  |  |  |  |  |
| Hansen Test P-Value | - | - | - | 0.809 |
| AR(1), AR(2), AR(3) P-Values | - | - | - | 0.059, 0.020, 0.091 |
| Observations | 136,385 | 136,385 | 107,278 | 108,093 |
| Individuals | 34,378 | 34,378 | 29,548 | 30,313 |
| R² (GMM: F) | 0.835 | 0.770 | 0.248 | F(77, 171) = 114.31 |
|  |  |  |  |  |
| *Panel B: Percentage Change in Mobility in Retail or Recreation* | | | | |
| Life Satisfaction | -0.0651*** | -0.1820*** | -0.1250*** | -0.0417 |
|  | (0.0148) | (0.0323) | (0.0393) | (0.0270) |
|  |  |  |  |  |
| Hansen Test P-Value | - | - | - | 0.578 |
| AR(1), AR(2), AR(3) P-Values | - | - | - | 0.029, 0.068, 0.901 |
| Observations | 136,385 | 136,385 | 107,278 | 108,093 |
| Individuals | 34,378 | 34,378 | 29,548 | 30,313 |
| R² (GMM: F) | 0.506 | 0.389 | 0.178 | F(77, 171) = 9.35 |
|  |  |  |  |  |
| *Panel C: Percentage Change in Mobility in Grocery and Pharmacy* | | | | |
| Life Satisfaction | -0.0688*** | -0.1960*** | -0.1310*** | -0.0214 |
|  | (0.0215) | (0.0440) | (0.0467) | (0.0273) |
|  |  |  |  |  |
| Hansen Test P-Value | - | - | - | 0.148 |
| AR(1), AR(2), AR(3) P-Values | - | - | - | 0.000, 0.000, 0.915 |
| Observations | 136,385 | 136,385 | 107,278 | 108,093 |
| Individuals | 34,378 | 34,378 | 29,548 | 30,313 |
| R² (GMM: F) | 0.480 | 0.326 | 0.184 | F(77, 171) = 5.72 |
|  |  |  |  |  |
| *Panel D: Percentage Change in Mobility in Parks* | | | | |
| Life Satisfaction | -0.2410*** | -0.5980*** | -0.4270*** | -0.2960*** |
|  | (0.0575) | (0.1130) | (0.1210) | -0.0722 |
|  |  |  |  |  |
| Hansen Test P-Value | - | - | - | 0.052 |
| AR(1), AR(2), AR(3) P-Values | - | - | - | 0.445, 0.000, 0.759 |
| Observations | 136,385 | 136,385 | 107,278 | 108,093 |
| Individuals | 34,378 | 34,378 | 29,548 | 30,313 |
| R² (GMM: F) | 0.341 | 0.168 | 0.045 | F(77, 171) = 15.39 |
|  |  |  |  |  |
| *Panel E: Percentage Change in Mobility in Transit* | | | | |
| Life Satisfaction | -0.1020*** | -0.2720*** | -0.1720*** | -0.1060*** |
|  | (0.0195) | (0.0374) | (0.0405) | (0.0265) |
|  |  |  |  |  |
| Hansen Test P-Value | - | - | - | 0.005 |
| AR(1), AR(2), AR(3) P-Values | - | - | - | 0.148, 0.000, 0.756 |
| Observations | 136,385 | 136,385 | 107,278 | 108,093 |
| Individuals | 34,378 | 34,378 | 29,548 | 30,313 |
| R² (GMM: F) | 0.450 | 0.182 | 0.133 | F(77, 171) = 9.16 |
|  |  |  |  |  |
| *Panel F: Percentage Change in Mobility in Workplaces* | | | | |
| Life Satisfaction | -0.0872*** | -0.2520*** | -0.1810*** | -0.0677 |
|  | (0.0159) | (0.0331) | (0.0438) | (0.0457) |
|  |  |  |  |  |
| Hansen Test P-Value | - | - | - | 0.349 |
| AR(1), AR(2), AR(3) P-Values | - | - | - | 0.256, 0.048, 0.388 |
| Observations | 136,385 | 136,385 | 107,278 | 108,093 |
| Individuals | 34,378 | 34,378 | 29,548 | 30,313 |
| R² (GMM: F) | 0.643 | 0.572 | 0.217 | F(77, 171) = 26.11 |
|  |  |  |  |  |
| Controls | Yes | Yes | Yes | Yes |
| Area Fixed Effects | Yes | Yes | Yes | Yes |
| Country Fixed Effects | Yes | Yes | Yes | Yes |
| Week-Day Fixed Effects | Yes | Yes | Yes | Yes |
| Week Fixed Effects | No | No | No | No |
| Individual Fixed Effects | No | Yes | Yes | Yes |
| See Study 2 in main text for model specifications.  Robust standard errors clustered at country time date level in parentheses.  *** p<0.01, ** p<0.05, * p<0.1 | | | | |

| *Table S.2.6: Life Satisfaction and Self-Reported Compliance (University College London Covid-19 Social Study, UK, Year 2020)  – No Person Restrictions* | | | | |
| --- | --- | --- | --- | --- |
|  | Static Panel Data Estimation | | | Dynamic Panel Data Estimation |
|  | Pooled OLS | FE | FD | Arellano-Bond |
|  | (1) | (2) | (3) | (4) |
| *Panel A: Number of Weekdays Staying Home* | | | | |
| Life Satisfaction | 0.0574*** | 0.0176*** | 0.0189*** | 0.0188*** |
|  | (0.0050) | (0.0044) | (0.0047) | (0.0041) |
|  |  |  |  |  |
| Hansen Test P-Value | - | - | - | 0.381 |
| AR(1), AR(2), AR(3) P-Values | - | - | - | 0.000, 0.000, 0.041 |
| Observations | 202,497 | 202,497 | 151,677 | 153,945 |
| Individuals | 50,218 | 50,218 | 37,615 | 43,408 |
| R² (GMM: F) | 0.082 | 0.008 | 0.006 | F(77, 43407) = 368.39 |
|  |  |  |  |  |
| *Panel B: Fully Isolating* | | | | |
| Life Satisfaction | -0.0005 | -0.0039*** | -0.0008* | 0.0007 |
|  | (0.0005) | (0.0005) | (0.0005) | (0.0006) |
|  |  |  |  |  |
| Hansen Test P-Value | - | - | - | 0.918 |
| AR(1), AR(2), AR(3) P-Values | - | - | - | 0.000, 0.000, 0.388 |
| Observations | 203,133 | 203,133 | 153,777 | 156,413 |
| Individuals | 50,030 | 50,030 | 37,874 | 43,577 |
| R² (GMM: F) | 0.073 | 0.030 | 0.011 | F(77, 43576) = 169.48 |
|  |  |  |  |  |
| *Panel C: Partially Isolating* |  |  |  |  |
| Life Satisfaction | 0.0008 | 0.0041*** | 0.0016** | 0.0009 |
|  | (0.0006) | (0.0008) | (0.0007) | (0.0007) |
|  |  |  |  |  |
| Hansen Test P-Value | - | - | - | 0.629 |
| AR(1), AR(2), AR(3) P-Values | - | - | - | 0.000, 0.000, 0.063 |
| Observations | 203,133 | 203,133 | 153,777 | 156,413 |
| Individuals | 50,030 | 50,030 | 37,874 | 43,577 |
| R² (GMM: F) | 0.055 | 0.036 | 0.015 | F(77, 43576) = 125.01 |
|  |  |  |  |  |
| *Panel D: Not Isolating* |  |  |  |  |
| Life Satisfaction | -0.0002 | -0.0001 | -0.0008 | -0.0013 |
|  | (0.0004) | (0.0006) | (0.0006) | (0.0004) |
|  |  |  |  |  |
| Hansen Test P-Value | - | - | - | 0.237 |
| AR(1), AR(2), AR(3) P-Values | - | - | - | 0.000, 0.000, 0.089 |
| Observations | 203,133 | 203,133 | 153,777 | 156,413 |
| Individuals | 50,030 | 50,030 | 37,874 | 43,577 |
| R² (GMM: F) | 0.125 | 0.130 | 0.045 | F(77, 43576) = 93.41 |
|  |  |  |  |  |
| *Panel E: Complying With Recommendations* | | | | |
| Life Satisfaction | 0.0142*** | 0.0032*** | 0.0017 | 0.0070 |
|  | (0.0013) | (0.0011) | (0.0012) | (0.0043) |
|  |  |  |  |  |
| Hansen Test P-Value | - | - | - | 0.157 |
| AR(1), AR(2), AR(3) P-Values | - | - | - | 0.116, 0.506, 0.330 |
| Observations | 209,534 | 209,534 | 162,038 | 163,643 |
| Individuals | 50,485 | 50,485 | 39,041 | 43,072 |
| R² (GMM: F) | 0.107 | 0.015 | 0.013 | F(78, 43071) = 119.20 |
|  |  |  |  |  |
| Controls | Yes | Yes | Yes | Yes |
| Area Fixed Effects | Yes | Yes | Yes | Yes |
| Country Fixed Effects | Yes | Yes | Yes | Yes |
| Week-Day Fixed Effects | Yes | Yes | Yes | Yes |
| Week Fixed Effects | No | No | No | No |
| Individual Fixed Effects | No | Yes | Yes | Yes |
| See Study 2 for model specifications and the Supplementary Materials for summary statistics.  Robust standard errors clustered at individual level in parentheses.  *** p<0.01, ** p<0.05, * p<0.1 | | | | |

| *Table S.2.7: Summary Statistics (University College London Covid-19 Social Study, UK, Year 2020)* | | | | | |
| --- | --- | --- | --- | --- | --- |
| Variable | Mean | Standard Deviation | Min. | Max. | Number of Observations |
|  |  |  |  |  |  |
| *Self-Reported Outcomes* |  |  |  |  |  |
| Number of Weekdays Staying Home | 3.1999 | 2.7353 | 0 | 7 | 131,088 |
| Fully Isolating | 0.0939 | 0.2917 | 0 | 1 | 127,538 |
| Partially Isolating | 0.8448 | 0.3621 | 0 | 1 | 127,538 |
| Not Isolating | 0.0613 | 0.2399 | 0 | 1 | 127,538 |
| Complying With Recommendations | 6.6250 | 0.6635 | 1 | 7 | 131,088 |
|  |  |  |  |  |  |
| *Observed Outcomes* |  |  |  |  |  |
| Percentage Change in Mobility in Residential Areas | 23.9003 | 4.2060 | 13 | 31 | 131,088 |
| Percentage Change in Mobility in Retail or Recreation | -72.3762 | 6.3819 | -93 | -36 | 131,088 |
| Percentage Change in Mobility in Grocery and Pharmacy | -28.7901 | 7.3647 | -69 | -6 | 131,088 |
| Percentage Change in Mobility in Parks | -13.2206 | 16.5106 | -71 | 21 | 131,088 |
| Percentage Change in Mobility in Transit | -61.7994 | 6.3808 | -76 | -36 | 131,088 |
| Percentage Change in Mobility in Workplaces | -62.5649 | 8.2447 | -83 | -40 | 131,088 |
|  |  |  |  |  |  |
| *Variables of Interest* |  |  |  |  |  |
| Life Satisfaction | 6.0283 | 2.2377 | 0 | 10 | 131,088 |
|  |  |  |  |  |  |
| *Controls* |  |  |  |  |  |
| Age | 53.9694 | 14.9891 | 18 | 96 | 131,088 |
| Is Female | 0.7236 | 0.4472 | 0 | 1 | 131,088 |
| Is Single, Never Married | 0.1526 | 0.3596 | 0 | 1 | 131,088 |
| Is Single, Divorced, or Widowed | 0.1468 | 0.3539 | 0 | 1 | 131,088 |
| Is in Relationship or Married but Living Apart | 0.0542 | 0.2264 | 0 | 1 | 131,088 |
| Is in Relationship or Married and Cohabitating | 0.6464 | 0.4781 | 0 | 1 | 131,088 |
| Is Asian | 0.0106 | 0.1024 | 0 | 1 | 131,088 |
| Is Black | 0.0036 | 0.0603 | 0 | 1 | 131,088 |
| Is Mixed Race, White | 0.0031 | 0.0560 | 0 | 1 | 131,088 |
| Is Mixed Race, Other | 0.0100 | 0.0997 | 0 | 1 | 131,088 |
| Is White | 0.9608 | 0.1940 | 0 | 1 | 131,088 |
| Is Chinese | 0.0028 | 0.0524 | 0 | 1 | 131,088 |
| Is Middle Eastern | 0.0018 | 0.0419 | 0 | 1 | 131,088 |
| Is Other Ethnic Group | 0.0045 | 0.0669 | 0 | 1 | 131,088 |
| Ethnicity: Prefer not to Say | 0.0027 | 0.0520 | 0 | 1 | 131,088 |
| Big-5: Openness | 5.1122 | 1.0886 | 1 | 7 | 131,088 |
| Big-5: Conscientiousness | 5.2714 | 0.9912 | 1 | 7 | 131,088 |
| Big-5: Extraversion | 4.2437 | 1.4359 | 1 | 7 | 131,088 |
| Big-5: Agreeableness | 5.1685 | 1.0168 | 1 | 7 | 131,088 |
| Big-5: Neuroticism | 3.7191 | 1.4480 | 1 | 7 | 131,088 |
| PHQ-9 | 5.7506 | 5.5191 | 0 | 27 | 131,088 |
| GAD-7 | 4.5056 | 4.9601 | 0 | 21 | 131,088 |
| Has no Qualification | 0.0304 | 0.1717 | 0 | 1 | 131,088 |
| Has O-Levels | 0.1157 | 0.3198 | 0 | 1 | 131,088 |
| Has Vocational Education | 0.0566 | 0.2311 | 0 | 1 | 131,088 |
| Has A-Levels | 0.1197 | 0.3246 | 0 | 1 | 131,088 |
| Has Undergraduate Degree | 0.4090 | 0.4917 | 0 | 1 | 131,088 |
| Has Postgraduate Degree | 0.2686 | 0.4432 | 0 | 1 | 131,088 |
| Is in School | 0.0022 | 0.0466 | 0 | 1 | 131,088 |
| Is in University | 0.0310 | 0.1733 | 0 | 1 | 131,088 |
| Is Self-Employed | 0.1018 | 0.3024 | 0 | 1 | 131,088 |
| Is Part-Time Employed | 0.1154 | 0.3195 | 0 | 1 | 131,088 |
| Is Full-Time Employed | 0.2908 | 0.4541 | 0 | 1 | 131,088 |
| Is Unable to Work Due to Disability | 0.0612 | 0.2397 | 0 | 1 | 131,088 |
| Is Home-Maker, Full-Time Parent | 0.0447 | 0.2066 | 0 | 1 | 131,088 |
| Is Unemployed | 0.0236 | 0.1517 | 0 | 1 | 131,088 |
| Is Retired | 0.3294 | 0.4700 | 0 | 1 | 131,088 |
| Annual Income < £16,000 | 0.1491 | 0.3562 | 0 | 1 | 131,088 |
| Annual Income £16,000-£29,999 | 0.2292 | 0.4203 | 0 | 1 | 131,088 |
| Annual Income £30,000-£59,999 | 0.3004 | 0.4585 | 0 | 1 | 131,088 |
| Annual Income £60,000-£89,999 | 0.1271 | 0.3331 | 0 | 1 | 131,088 |
| Annual Income £90,000-£119,999 | 0.0538 | 0.2256 | 0 | 1 | 131,088 |
| Annual Income > £120,000 | 0.0380 | 0.1913 | 0 | 1 | 131,088 |
| Income: Prefer not to Say | 0.1023 | 0.3030 | 0 | 1 | 131,088 |
| Lives in House | 0.8008 | 0.3994 | 0 | 1 | 131,088 |
| Lives in Shared House | 0.0142 | 0.1184 | 0 | 1 | 131,088 |
| Lives in Flat | 0.1412 | 0.3482 | 0 | 1 | 131,088 |
| Lives in Student Halls | 0.0019 | 0.0434 | 0 | 1 | 131,088 |
| Lives in Residential Home | 0.0031 | 0.0560 | 0 | 1 | 131,088 |
| Lives in Other | 0.0388 | 0.1931 | 0 | 1 | 131,088 |
| Has 1 Room | 0.0115 | 0.1068 | 0 | 1 | 131,088 |
| Has 2 Rooms | 0.0428 | 0.2023 | 0 | 1 | 131,088 |
| Has 3 Rooms | 0.0999 | 0.2999 | 0 | 1 | 131,088 |
| Has 4 Rooms | 0.1493 | 0.3564 | 0 | 1 | 131,088 |
| Has 5 Rooms | 0.2005 | 0.4004 | 0 | 1 | 131,088 |
| Has 6 Rooms | 0.1893 | 0.3918 | 0 | 1 | 131,088 |
| Has 7 Rooms | 0.1384 | 0.3453 | 0 | 1 | 131,088 |
| Has 8 Rooms | 0.0888 | 0.2844 | 0 | 1 | 131,088 |
| Has 9 Rooms | 0.0441 | 0.2054 | 0 | 1 | 131,088 |
| Has 10+ Rooms | 0.0354 | 0.1848 | 0 | 1 | 131,088 |
| Number of Adults in Household | 1.0645 | 0.9035 | 0 | 10 | 131,088 |
| Number of Children in Household | 0.3524 | 0.7755 | 0 | 10 | 131,088 |
| Number of Close Friends | 4.8641 | 3.1205 | 0 | 10 | 131,088 |
| Frequency of Socialising Face-to-Face | 2.9021 | 1.1430 | 1 | 5 | 131,088 |
| Knowledge About Covid-19 | 5.4450 | 1.0459 | 1 | 7 | 131,088 |
| Confidence in Government | 4.4423 | 1.6507 | 1 | 7 | 131,088 |
| Confidence in National Health Service | 5.1536 | 1.3643 | 1 | 7 | 131,088 |
| Average Daily Number of New Covid-19 Cases | 4,329.4 | 1,390.8 | 665 | 8,719 | 131,088 |
| Average Daily Number of New Covid-19 Deaths | 665.8 | 301.1 | 35 | 1,172 | 131,088 |
| Lives in City | 0.3220 | 0.4672 | 0 | 1 | 131,088 |
| Lives in Large Town | 0.1686 | 0.3744 | 0 | 1 | 131,088 |
| Lives in Small Town | 0.2535 | 0.4350 | 0 | 1 | 131,088 |
| Lives in Village | 0.2076 | 0.4056 | 0 | 1 | 131,088 |
| Lives in Hamlet | 0.0276 | 0.1638 | 0 | 1 | 131,088 |
| Lives in Isolated Dwelling | 0.0207 | 0.1423 | 0 | 1 | 131,088 |
| England | 0.8080 | 0.3939 | 0 | 1 | 131,088 |
| Wales | 0.1101 | 0.3131 | 0 | 1 | 131,088 |
| Scotland | 0.0713 | 0.2573 | 0 | 1 | 131,088 |
| Northern Ireland | 0.0106 | 0.1024 | 0 | 1 | 131,088 |

**Additional Study**

**Study: Cross-Sectional Evidence on Regional Life Satisfaction and Observed Regional Compliance**

Throughout the world, in the early months of 2020, different countries (and different regions within countries) introduced lockdowns to contain the spread of Covid-19. We exploit this regional variation within countries across the world at different points in time in an additional empirical investigation, looking, in particular, at the predictive power of prior life satisfaction for subsequent compliance with preventive health measures.

**Data and Methods.** To study whether past regional life satisfaction predicts observed regional compliance with preventive health behaviours during Covid-19 lockdowns, we use cross-sectional data from the Gallup World Poll, a nationally representative survey which is conducted annually in more than 160 countries worldwide and which includes data on about 1,000 respondents’ life satisfaction alongside a wide range of individual, household, and regional characteristics in each country. We again merge these data with two other datasets at the regional level: the Oxford COVID-19 Government Response Tracker [54], which includes daily data on the stringency of lockdown measures (at the country level), as well as confirmed cases and deaths (at the regional level); and the Google COVID-19 Community Mobility Reports, which include daily regional data on smartphone-recorded geographical movement to various areas via Google Maps, which we take as objective measures of compliance behaviour [66, 67].

Our outcomes are daily percentage changes in smartphone-recorded geographical visits to (or time spent in) residential, retail or recreation, grocery or pharmacy, parks, transit, and workplace areas. These daily changes are compared to an average baseline day between January 3 and February 6, 2020. Note that residential areas refer to all areas that Google Maps classifies as “residential”, not only the actual places of residence of individuals. That said, it is possible that it includes time spent in other individuals’ places of residence (which too are likely to be located in what Google Maps classifies as residential areas). Unfortunately, the data do not allow us to empirically exclude such cases. However, we believe that it is rather unlikely that systematic changes in cohabitation behaviour are driving our results. Still, our findings should be interpreted with this caveat in mind, in particular that increased time spent in residential areas may also reflect a substitution effect if spent socially, hence contributing to a potential overestimate of our association between compliance behaviour and life satisfaction.

Our variable of interest is again life evaluation (i.e. the *Cantril ladder*; see [55]). It is obtained from a single-item eleven-point Likert scale which asks respondents to imagine themselves on a ladder with steps numbered from zero at the bottom to ten at the top, where zero represents the worst possible and ten the best possible life. Recall that, in practice, life evaluation and life satisfaction are often seen as equivalent, so in what follows we refer to it as *life satisfaction* for simplicity.

We regress regional-level geographical mobility during Covid-19 lockdowns in 2020 on regional-level life satisfaction in 2019:

*compliance_rt_ = a + β_1_satisfaction_r2019_ + X_rt_β_2_' + η_wd_ + η_w_ + μ_c_ + ε_rt_* (1)

where *compliance_rt_* is the outcome in region *r* on date *d*, *satisfaction_r2019_* is the predicted life satisfaction in region *r* in year *2019*, *X* is a vector of controls, and *η_wd_* are weekday, *η_w_* calendar week, and *μ_c_* country fixed effects. The controls include indices of local economic confidence, unemployment, law and order, and social life, and mean regional household income from the Gallup World Poll, as well as the log daily numbers of confirmed Covid-19 cases and deaths from the Oxford COVID-19 Government Response Tracker. Predicted life satisfaction is obtained from an auxiliary model that regresses a respondent’s life satisfaction on age, gender, marital status, education, employment status, log household income, the number of adults and children in the household, and the degree of urbanisation. The model is estimated using OLS, with robust standard errors clustered at the regional level (clustering at the interview date level yields similar results).

Our sample is restricted to observations where the stringency of lockdown measures is equal to or greater than 72.48, to ensure comparability to our other studies. A stringency of 72.48 corresponds to the stringency observed during the first “hard” UK lockdown period, which is the focus of our main Study 2. Our results continue to hold when lifting these restrictions (see Table S.3.2 below). Our sample includes data on between 48,520 and 50,966 adult respondents (depending on outcome) from 892 regions in 49 countries. Table S.3.3 shows summary statistics.

Although we are controlling for observables (e.g. age, employment status, log household income, the degree of urbanisation, etc.) when predicting average regional life satisfaction in our auxiliary model, as well as for indices of local economic confidence, unemployment, law and order, social life, and mean regional household income when regressing regional compliance with Covid-19 measures on predicted average regional life satisfaction in our main model, our estimation relies on between-regional variation in life satisfaction and compliance behaviour, due to the cross-sectional nature of our data. That said, we are unable to control for time-invariant unobservable heterogeneity or time-invariant, potentially omitted observables at both individual and regional level. Our obtained coefficients should, therefore, strictly be interpreted as associations and upper-bound estimates of the true association between compliance behaviour and life satisfaction.

**Findings.** We find that higher regional-level life satisfaction in 2019 is associated with higher regional-level compliance during Covid-19 lockdowns in 2020 (see Table S.3.1 below). More specifically, a one-point increase in life satisfaction in 2019 is associated with decreases between ten percent and 22% in visits to retail or recreation, grocery or pharmacy, parks, transit, and workplaces. On the contrary, geographical mobility in residential areas is associated with increases by about six percent.

**Limitations.** Our cross-sectional study of observed regional compliance with Covid-19 measures and regional life satisfaction has several caveats. First, although the Google COVID-19 Community Mobility Reports have been routinely used to objectively measure compliance behaviour in cross-country and cross-regional studies (see Bargain and Amininjov (2020, Journal of Public Economics) or Nouvellet et al. (2021, Nature Communications), for example), we are not aware of any study that looks at whether these data are equally valid and reliable across countries and regions. To the extent that populations using Google Maps may differ by country, and to the extent that there may be differences in how Google Maps classifies certain areas between countries, we control for such differences by including country fixed effects. Second, our measure of geographical regions is rather coarse, pertaining to the first administrative layer below the country level (e.g. for the US, these are the 50 States). Unfortunately, this is the only administrative layer at which we can conduct a merge between our Gallup World Poll data and the Google COVID-19 Community Mobility Reports. To avoid any non-uniform mitigation policies between regions within a country as well as between countries, we restrict our final sample to observations where the stringency of lockdown measures was equal to or greater than 72.48 (the stringency observed during the strict lockdown period in the UK), i.e. we only look at regions that showed a homogeneous policy response. Third, since we are conducting an analysis at the regional level, our findings may be subject to the ecological fallacy, i.e. we may be erroneously attributing regional behaviour to individual behaviour, which may not be true. Finally, there may be unobservable differences between regions that we do not have data on and cannot fully control for (e.g. we cannot control for region fixed effects as these would be collinear with our variable of interest, i.e. life satisfaction in 2019). Our findings should, therefore, be interpreted with caution.

| *Table S.3.1: Life Satisfaction and Observed Compliance (Gallup World Poll, 892 Regions, 49 Countries, Years 2019 to 2020)* | | | | | | | | | | |
| --- | --- | --- | --- | --- | --- | --- | --- | --- | --- | --- |
|  | | Percentage Change in Mobility During Lockdown in | | | | | | | | |
|  | | Residential Areas | | Retail or Recreation | | Grocery, Pharmacy | | Parks | Transit | Workplaces |
|  | | (1) | | (2) | | (3) | | (4) | (5) | (6) |
| *Panel A: Fixed Effects Only* | | | | | | | | | | |
| Life Satisfaction in 2019 | | 5.2021*** | | -11.2052*** | | -9.4766*** | | -17.8317** | -21.3671*** | -10.0029*** |
|  | | (1.0534) | | (3.2859) | | (2.2898) | | (8.1332) | (4.2677) | (2.3033) |
|  | |  | |  | |  | |  |  |  |
| Controls | | No | | No | | No | | No | No | No |
| Country Fixed Effects | | Yes | | Yes | | Yes | | Yes | Yes | Yes |
| Week-Day Fixed Effects | | Yes | | Yes | | Yes | | Yes | Yes | Yes |
| Week Fixed Effects | | Yes | | Yes | | Yes | | Yes | Yes | Yes |
|  | |  | |  | |  | |  |  |  |
| Observations | | 84,137 | | 122,034 | | 116,625 | | 64,697 | 85,246 | 169,785 |
| R² | | 0.582 | | 0.645 | | 0.520 | | 0.388 | 0.512 | 0.531 |
|  | |  | |  | |  | |  |  |  |
| *Panel B: Covariates and Fixed Effects* | | | | | | | | | | |
| Life Satisfaction in 2019 | | 5.7800*** | | -12.3900*** | | -10.0100*** | | -19.9400** | -22.2500*** | -10.7800*** |
|  | | (1.0110) | | (3.0390) | | (2.2020) | | (8.2450) | (3.9180) | (2.3480) |
|  | |  | |  | |  | |  |  |  |
| Controls | | Yes | | Yes | | Yes | | Yes | Yes | Yes |
| Country Fixed Effects | | Yes | | Yes | | Yes | | Yes | Yes | Yes |
| Week-Day Fixed Effects | | Yes | | Yes | | Yes | | Yes | Yes | Yes |
| Week Fixed Effects | | Yes | | Yes | | Yes | | Yes | Yes | Yes |
|  | |  | |  | |  | |  |  |  |
| Observations | | 83,278 | | 119,672 | | 114,756 | | 63,868 | 84,139 | 166,435 |
| R² | | 0.605 | | 0.657 | | 0.539 | | 0.401 | 0.534 | 0.535 |
| Robust standard errors clustered at regional level in parentheses. | | | | | | | | | | |
| *** p<0.01, ** p<0.05, * p<0.1 | | | | | | | | | | |
| Table S.3.2: Life Satisfaction and Observed Compliance (Gallup World Poll, 892 Regions, 49 Countries, Years 2019 to 2020) – No Stringency Restrictions | | | | | | | | | | |
|  | Percentage Change in Mobility During Lockdown in | | | | | | | | | |
|  | Residential Areas | | Retail or Recreation | | Grocery, Pharmacy | | Parks | | Transit | Workplaces |
|  | (1) | | (2) | | (3) | | (4) | | (5) | (6) |
| *Panel A: Fixed Effects Only* | | | | | | | | | | |
| Life Satisfaction in 2019 | 3.2513*** | | -5.8350*** | | -5.2410*** | | -9.1748* | | -13.3607*** | -6.8645*** |
|  | (0.6011) | | (2.0750) | | (1.3809) | | (4.9947) | | (2.7547) | (1.3851) |
|  |  | |  | |  | |  | |  |  |
| Controls | No | | No | | No | | No | | No | No |
| Country Fixed Effects | Yes | | Yes | | Yes | | Yes | | Yes | Yes |
| Week-Day Fixed Effects | Yes | | Yes | | Yes | | Yes | | Yes | Yes |
| Week Fixed Effects | Yes | | Yes | | Yes | | Yes | | Yes | Yes |
|  |  | |  | |  | |  | |  |  |
| Observations | 166,249 | | 247,740 | | 239,657 | | 121,754 | | 155,977 | 299,456 |
| R² | 0.781 | | 0.757 | | 0.506 | | 0.314 | | 0.641 | 0.771 |
|  |  | |  | |  | |  | |  |  |
| *Panel B: Covariates and Fixed Effects* | | | | | | | | | | |
| Life Satisfaction in 2019 | 4.6182*** | | -9.0657*** | | -7.3103*** | | -14.5853** | | -18.2893*** | -9.0048*** |
|  | (0.7682) | | (2.5309) | | (1.7830) | | (6.6373) | | (3.2203) | (1.7502) |
|  |  | |  | |  | |  | |  |  |
| Controls | Yes | | Yes | | Yes | | Yes | | Yes | Yes |
| Country Fixed Effects | Yes | | Yes | | Yes | | Yes | | Yes | Yes |
| Week-Day Fixed Effects | Yes | | Yes | | Yes | | Yes | | Yes | Yes |
| Week Fixed Effects | Yes | | Yes | | Yes | | Yes | | Yes | Yes |
|  |  | |  | |  | |  | |  |  |
| Observations | 125,644 | | 184,652 | | 178,226 | | 91,587 | | 120,175 | 234,308 |
| R² | 0.764 | | 0.776 | | 0.607 | | 0.392 | | 0.634 | 0.751 |
| Robust standard errors clustered at regional level in parentheses. | | | | | | | | | | |
| *** p<0.01, ** p<0.05, * p<0.1 | | | | | | | | | | |

| *Table S.3.3: Summary Statistics (Gallup World Poll, 892 Regions, 49 Countries, Years 2019 to 2020)* | | | | |  |
| --- | --- | --- | --- | --- | --- |
| Variable | Mean | Standard Deviation | Minimum | Maximum | Number of Observations |
|  |  |  |  |  |  |
| *Observed Outcomes* |  |  |  |  |  |
| Percentage Change in Mobility During Lockdown in… |  |  |  |  |  |
| …Residential Areas | 17.4990 | 6.6816 | -11 | 54 | 83,278 |
| …Retail or Recreation | -43.1058 | 21.1181 | -98 | 35 | 81,485 |
| …Grocery, Pharmacy | -15.3415 | 18.7978 | -97 | 124 | 80,299 |
| …Parks | -9.2830 | 49.1642 | -97 | 413 | 55,658 |
| …Transit | -43.1671 | 24.2223 | -100 | 77 | 64,564 |
| …Workplaces |  |  |  |  |  |
|  |  |  |  |  |  |
| *Variables of Interest* |  |  |  |  |  |
| Life Satisfaction in 2019 | 6.5590 | 0.5210 | 2.7121 | 7.4439 | 83,278 |
|  |  |  |  |  |  |
| *Controls* |  |  |  |  |  |
| Index of Local Economic Confidence | 99.2657 | 7.0997 | -50 | 100 | 83,278 |
| Index of Unemployment | 0.5705 | 0.4950 | 0 | 1 | 83,278 |
| Index of Social Life | 99.8667 | 2.5781 | 50 | 100 | 83,278 |
| Index of Law and Order | 99.8247 | 2.0862 | 75 | 100 | 83,278 |
| Number of New Covid-19 Cases | 430,472.6 | 426,939.2 | 31 | 1,283,929 | 83,278 |
| Number of New Covid-19 Deaths | 23,003.8 | 25,274.1 | 1 | 77,180 | 83,278 |
